# Shifting seas, shifting boundaries: Dynamic marine protected area designs for a changing climate

**DOI:** 10.1371/journal.pone.0241771

**Published:** 2020-11-10

**Authors:** Tim Cashion, Tu Nguyen, Talya ten Brink, Anne Mook, Juliano Palacios-Abrantes, Sarah M. Roberts

**Affiliations:** 1 Fisheries Economics Research Unit, Institute for the Oceans and Fisheries, University of British Columbia, Vancouver, Canada; 2 Department of Applied Economics, Oregon State University, Corvallis, OR, United States of America; 3 Greater Atlantic Regional Fisheries Office, National Marine Fisheries Service, National Oceanic and Atmospheric Administration, Gloucester, MA, United States of America; 4 Department of Sociology & Anthropology, Nazarbayev University, Nur Sultan, Kazakhstan; 5 Changing Ocean Research Unit, Institute for the Oceans and Fisheries, University of British Columbia, Vancouver, Canada; 6 Marine Geospatial Ecology Lab, Nicholas School of the Environment and Earth Sciences, Duke University, Durham, NC, United States of America; Swedish University of Agricultural Sciences and Swedish Institute for the Marine Environment, University of Gothenburg, SWEDEN

## Abstract

Marine protected areas (MPAs) are valuable tools for marine conservation that aim to limit human impacts on marine systems and protect valuable species or habitats. However, as species distributions shift due to ocean warming, acidification, and oxygen depletion from climate change, the areas originally designated under MPAs may bear little resemblance to their past state. Different approaches have been suggested for coping with species on the move in conservation. Here, we test the effectiveness of different MPA designs, including dynamic, network, and different directional orientations on protecting shifting species under climate change through ecosystem modeling in a theoretical ecosystem. Our findings suggest that dynamic MPAs may benefit some species (e.g., whiting and anchovy) and fishing fleets, and these benefits can inform the design or adaptation of MPAs worldwide. In addition, we find that it is important to design MPAs with specific goals and to account for the effects of released fishing pressure and species interactions in MPA design.

## Introduction

Marine protected areas (MPAs) are among the most popular types of marine spatial planning strategies to protect species and habitats from harmful human activities [1]. MPA designs vary in shape and size, with some incorporating connected networks of MPAs. Positive social-ecological impacts of MPAs within their boundaries include increasing fish biomass, density, and size, maintaining species diversity, supporting food production, and providing aesthetic, recreational, and spiritual values [2, 3]. Some of these benefits have been shown to also occur outside of the MPAs boundaries, with the spillover (i.e., positive net migration of fish from no-take areas to the surrounding areas outside the MPA) of adult fish biomass occurring in waters up to two kilometers away, subsequently increasing fisheries yield in surrounding areas [4]. These potential fisheries benefits can lead to increased efficiency of fishing at the edges of MPAs due to higher concentrations of fish biomass, partially compensating for the lost fisheries revenue from the closed area. However, not all of these benefits are shared equitably and often lead to positive outcomes for some users and negative outcomes for others [5–7], necessitating a balance of trade-offs between different users.

There is vast evidence that marine species are shifting their distributions due to climate change around the world [8–12]. Moreover, modeling exercises suggest such shifts will continue [13–17], even with strong mitigation of greenhouse gases such as under the Paris Agreement [18]. Given that most MPAs have static locations and boundaries, MPAs may lose their function to protect specific habitats or species on the move under climate change [19, 20], which undermines the future benefits of MPAs and their ability to meet conservation goals [21]. Thus, management needs to explicitly consider climate change to protect species with shifting distributions [6, 21].

This paper responds to gaps in the MPA literature by examining the biological and economic effects of varied MPA designs on protecting functional groups as they shift their distributions under climate change [21–23]. While this has been identified as an area of necessary research, the authors are not aware of any modeling study on the effects of climate change on multiple MPA designs. Our aim was to explore the theoretical benefits of dynamic MPAs to respond to the effects of climate change through an ecosystem modeling approach. We evaluated these benefits in terms of three different outcomes: biomass, catch, and fisheries revenues. These outcomes are relevant measures of success of the MPA [24] and for a main group of resource users adjacent to many MPAs [25]. Therefore, this study had two central aims: i) to determine how the outcomes of MPAs vary under climate change, and ii) to evaluate how different MPA designs (static vs. dynamic, network vs. individual) perform under climate change.

## Methods

To undertake this analysis, we used a spatially explicit model in Ecospace, the spatial form of the ecosystem modeling software Ecopath with Ecosim [26]. Ecospace models begin with the parameterization of a static mass-balanced model (Ecopath), modified by a temporal scenario (Ecosim), and finally spatialized to a certain habitat (Ecospace). This software allows for the exploration of different MPA management strategies that incorporate ecological and social-economic criteria [27]. We adapted the theoretical ecosystem of ‘Anchovy Bay’ for our analysis [28]. This choice was made based on the finding that there are few developed Ecospace models due to high data requirements [29]. To incorporate the changing nature of the ecosystems, we assessed our proposed MPA designs under projected sea surface temperature change as a climate change driver. This simulation lends itself well to a theoretical ecosystem where we can ignore the uncertainty of ecosystem model parameterization in favor of separating out the effects of climate change and MPA design. We parameterized the model to represent the Anchovy Bay ecosystem in 2000 and allowed the model to run for 100 years (between 2000 and 2100). We calculated mean biomass, catch and fisheries revenue considering the last 10 years of the projection (between 2090 and 2100).

**Fig 1:**
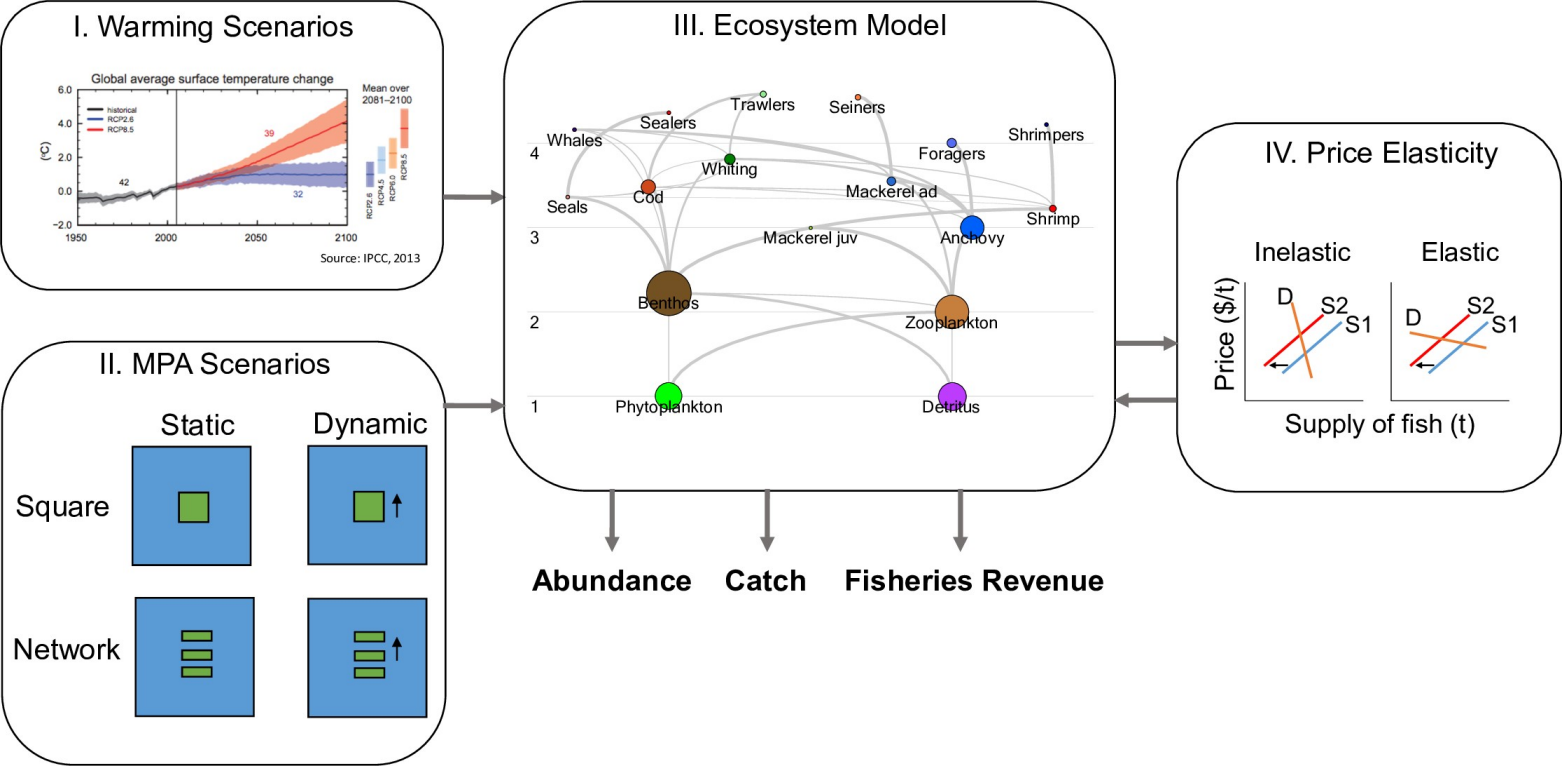
Conceptual figure of methods used for this study. We chose the warming scenario (Visual from IPCC, 2019), that mimics a future under RCP 8.5, and fit it with MPA designs to a spatialized ecosystem model. The ecosystem model includes predator-prey relationships between functional groups including fishing fleets. This model incorporated price elasticity when estimating fishing fleet dynamics. The outputs of the model are abundance, catch, and fisheries revenue in each time step for each functional group and fishing fleet.

We focused on two main MPA designs that have been identified as potential solutions to the conservation of species on the move: dynamic and network MPAs [21, 20]. We tested whether MPAs continue to have benefits under climate change (under 4°C sea surface temperature increase), or whether the negative effects of climate change can be reduced by certain MPA designs (Fig 1).

### Model design

The baseline model parameters and food web structure of the Anchovy Bay Ecospace model were adopted from [28]. The spatial layout of the ecosystem is a 20 by 20 grid (400 total cells), where each cell represents a 20 km by 20 km square. This model is made up of 11 functional groups, one of which has two age classes (juveniles and adults; Table 1). The input parameters of the Ecopath model can be found in S1 Table and the diet matrix in S2 Table. Of the 11 functional groups, six are targeted by fishing fleets. There are five fishing fleets that correspond to these targeted groups (Table 1). In this model, the functional groups’ populations are distributed based on the presence of prey and their temperature preference. In addition, some functional groups have the physical ability to travel farther than other functional groups and therefore the results of our modeling are specific to a functional groups’ ability to move as well as its temperature preferences. In this modeling framework, the ability to move is termed ‘dispersal.’ The dispersal values used in this model are based on a previous study that covered most of the functional groups in this ecosystem (S3 Table; [30]). Due to the theoretical nature of our modeling as well as a lack of data availability for the creation of spatially explicit ecosystem models that can incorporate climate change effects, we used an adapted model from a hypothetical ecosystem to test our hypotheses.

**Table 1:** Functional groups and corresponding fishing fleets included in the ecosystem model.

Functional group	Fishing fleet
Anchovy	Foragers
Benthos	
Cod	Trawlers
Detritus	
Mackerel (adult)	Seiners
Mackerel (juvenile)	
Phytoplankton	
Seals	Sealers
Shrimp	Shrimpers
Whales	
Whiting	Trawlers
Zooplankton	

Each Ecopath model starts in a mass-balanced steady-state. Simulating the ecosystem over time allows the functional groups’ populations to expand and decline based on their suitability to the ecosystem and the relative abundance of their prey and predators. Thus, our model takes into account predator-prey interactions over space and time. In addition to these ecological dynamics, fishing effort is spatially distributed based on a cost model that incorporates distance from the fishing port and the price for their target organism where fleets are dynamically driven to maximize their profits, and limited in expansion and contraction of the fleet size based on capital investment rates.

The model was adapted from the original in the following ways: incorporation of climate change impacts through forcing functions, inclusion of a north-south sea surface temperature gradient, and the removal of habitat features to not constrain functional groups. The remaining predator-prey dynamics between functional groups (including fisheries) were maintained.

We ran the model to simulate the ecosystem dynamics for a study period of 100 years. The model was constructed to compare the results of different MPA designs under climate change. We adopted an approximation for climate change effects on sea surface temperature based on the results of the Intergovernmental Panel on Climate Change (IPCC) estimates [31]. This estimate broadly encompasses a 4°C change in sea surface temperature by the end of the 21st century (2100). We incorporated this estimate in the ecosystem model through forcing functions that were applied to consumers and producers and were assumed to affect their ability to find prey (i.e., search rate). This is a common method for implementing climate change effects in Ecosim models to influence the survival of a functional group based on their temperature preferences. The result of modifying the search rate of a functional group by temperature preference is that as temperature moves farther away from a functional group’s optimal range, they are less effective at finding prey and thus more vulnerable to predation.

We defined environmental response parameters for species based on depth and temperature preferences. We then applied the forcing function of changes to sea surface temperature to the functional group specific response for the appropriate climate change scenario. To mimic a temperature gradient at a small scale (assuming a Northern Hemisphere ecosystem), we created a temperature gradient in the spatial ecosystem with colder temperatures in the ‘northern’ cells and warmer temperatures in the ‘southern’ cells. Therefore, functional groups can relocate amongst this spatial grid based on their thermal preferences.

To estimate fisheries revenues, the model uses the first-sale prices for different functional groups multiplied by their catch amount in each year. We used the defaults price values from the original Ecopath model [28] (S4 Table). The price elasticity of supply, the response of prices to increases or decreases in supply, was incorporated into the models based on known price elasticities of similar species/commercial groups. The values were sourced from a synthesis of the same product elasticity [32] and an average was taken where multiple values were reported for a functional group (S5 Table). This average was applied within the ecosystem model so that fishing effort behavior would respond to changes in prices and thus profitability of their fishing operations. Incorporating price elasticity allows a relatively realistic response of fishers changing incentives and behavior when coupled with distance-based fishing costs.

More details on the model parameterization can be found in the (S1 File). We used a scenario with no MPA as the baseline scenario for all simulations. Then, we applied alternative scenarios with the climate change effects and different MPA designs.

### MPA designs

We developed six MPA designs; four static MPAs (Square, Narrow Vertical, Narrow Horizontal and Network) to compare with two dynamic MPA designs (Square Shifting and Network Shifting) under climate change. (S2 Fig). The two dynamic MPAs have the same dimensions as their static counterparts, while moving by one cell height (20 km) every 20 years (S3 Fig). This rate of movement may be conservative as current estimates are between 15.5 km/decade and 25.6 km/decade for low and high-emissions scenarios, respectively [33]. We differentiate between the Static Horizontal and Static Vertical MPAs as we hypothesize that vertically oriented MPAs will be more likely to benefit species as they shift poleward due to climate change [8, 9]. This differentiation is relevant to include because the vertical orientation could fulfill similar roles to networks of protected areas that span across latitudinal gradients as species shift poleward under climate change [34, 35]. We did not include dynamic counterparts to these as the hypothesized goal of the Static Vertical MPA is to cover a wide range of temperature gradients providing protection as species migrate poleward. All designs with MPAs had the same amount of spatial area closed (e.g., 42 out of 400 cells, roughly 10% of the ecosystem), and visuals of the MPA designs can be found in the (S2 and S3 Figs).

### Statistics

We fit linear regression models to evaluate differences in aggregate levels of biomass, catch, and revenue between various MPA designs and the baseline no MPA scenario. We present our results comparing the average values of the present-day ecosystem (years 0–10) to the end of the century ecosystem (years 90–100).

## Results

**Fig 2:**
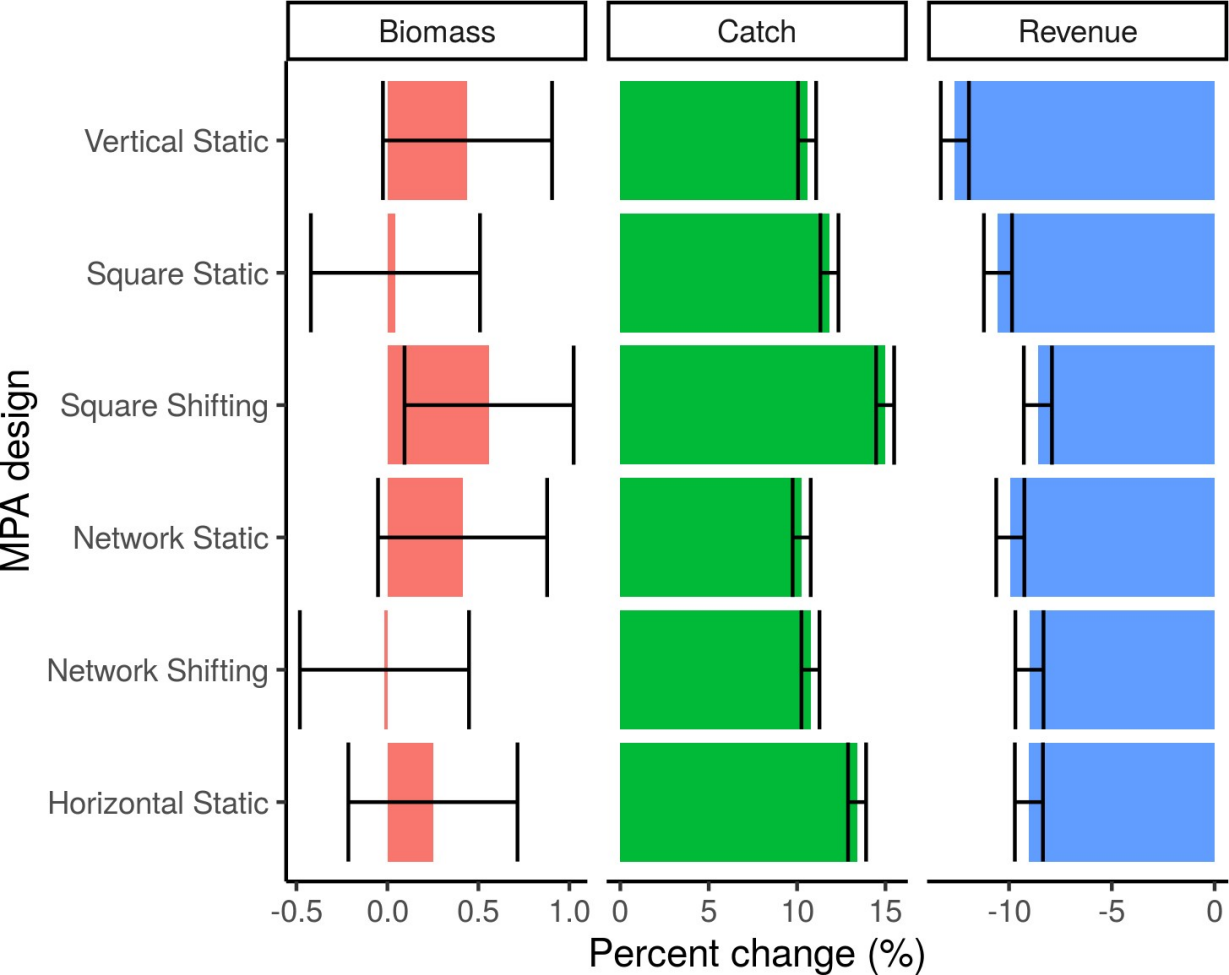
Percent change from the no MPA scenario at the end of the 21^st^ century under 4°C warming. All scenarios are significantly different in terms of catch and revenue from the no MPA scenario. Error bars represent 95% confidence intervals.

**Fig 3:**
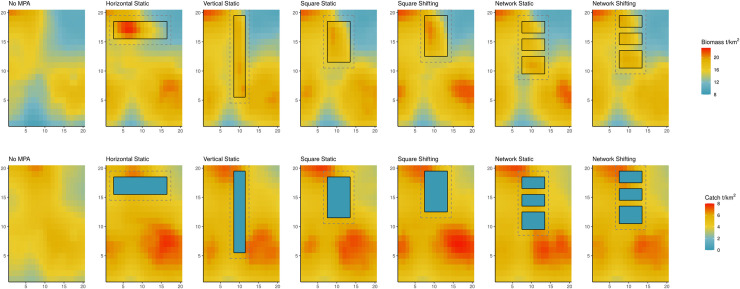
Biomass (t/km^2^) and catch (t/km^2^) of fished functional groups at the end of the 21^st^ century (years 90–100).

Our results suggest that there is a significant difference in biomass in only one MPA design scenario, Square Shifting, compared to the no MPA scenario (Fig 2, S6 Table); however, the magnitude of this difference in biomass is minimal (<5% difference in biomass under all MPA designs) (S7 Table). Catches are slightly higher (mean value of 12%) under all MPA scenarios (p-values < 0.001) than under the no MPA scenario (S8 Table). No single MPA design significantly outperforms the others in terms of both biomass and catch. Even with higher catches, revenue is significantly lower (mean value of -10%, p-values < 0.001) under all MPA scenarios than under the no MPA scenario (Fig 2) due to a combination of price elasticity and lower-value species being caught. Overall, there is strong variation of catches by species under different MPA designs, especially in the Network designs; however, the changes in catches between MPA designs at the edges of MPAs are minimal when aggregated across groups (Fig 3).

When comparing the dynamic MPAs to their static counterparts, we see that the Square Shifting MPA outperforms the Square Static MPA on all aggregate measures. The Network MPAs (Network Static and Network Shifting) perform similarly on all aggregate measures. The Square Shifting MPA compared to the Static Square MPA has significantly higher catches (15% compared to 11.8%, p-value < 0.001), revenue (-8.6% compared to -10.5%, p-value < 0.001), and biomass (0.6% compared to 0.0%, p-value < 0.001). In contrast, the network MPAs have <1% difference between their relative performance when compared against the no MPA scenario and their 95% confidence intervals overlap for all of their measures (Fig 2). Overall, the Square Shifting MPA also outperforms both network MPA designs significantly in terms of total catch, revenue, and biomass (Fig 2, S7–S9 Tables).

**Fig 4:**
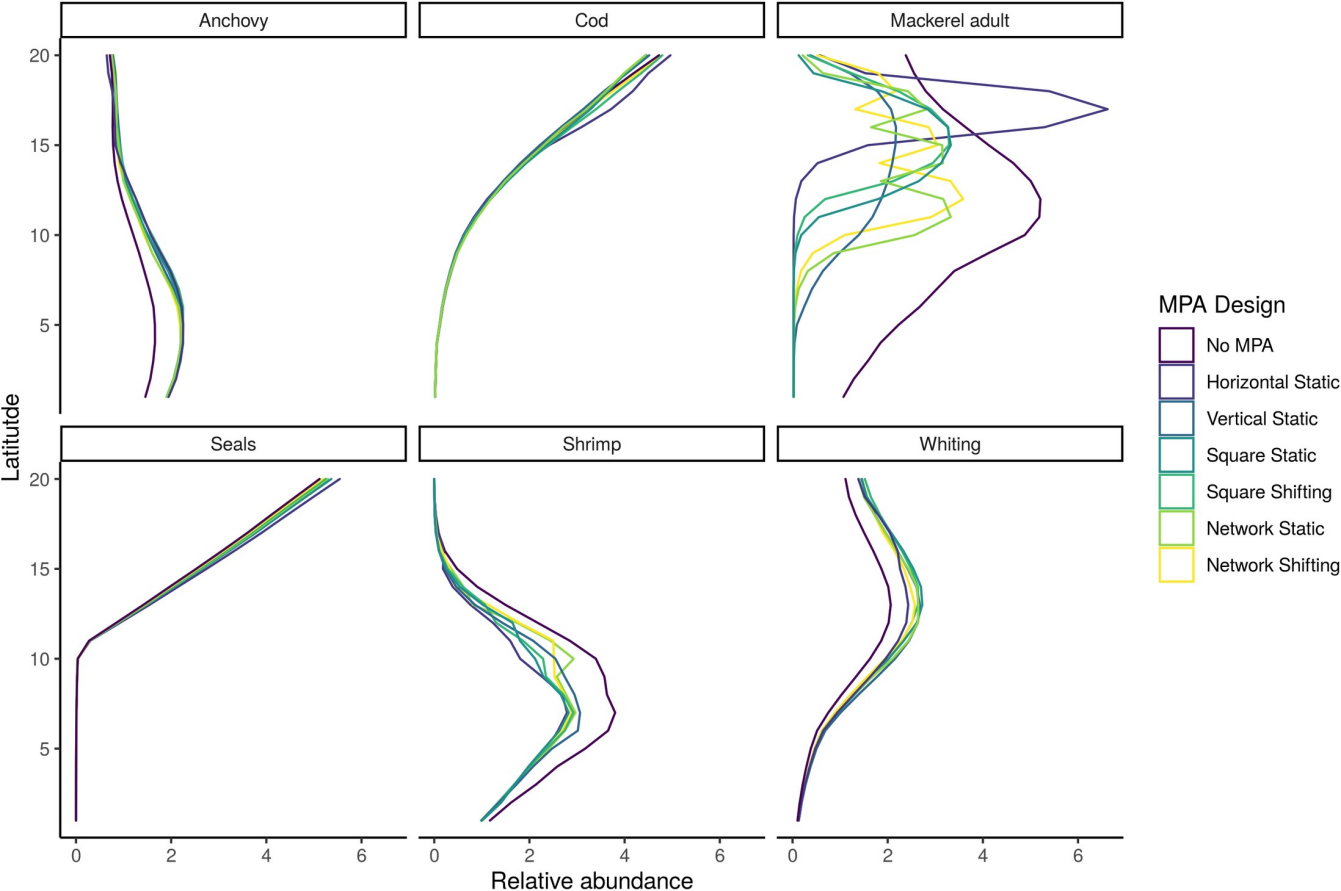
Normalized biomass of fished functional groups at the end of the 21^st^ century (years 90–100) by latitude.

We observe the change in species ranges through their average biomass at different latitudes in our ecosystem (Fig 4). For many fished species, the introduction of the MPAs does not fundamentally alter the species ranges. However, for adult mackerel, the effect of introducing an MPA is dramatic, shifting the range to almost solely within the MPA areas regardless of design (Fig 4 and S11 Fig). Comparing the Static Vertical and Horizontal MPAs, most functional groups’ biomass are heavily concentrated in the ‘northern’ latitudes in the horizontal MPA, but more evenly distributed in the Vertical MPA scenario. When comparing the Square designs to the Network designs (Static and Shifting), the Network designs have higher biomass levels in the Southern ranges than the Square designs.

### Species-specific results

**Fig 5:**
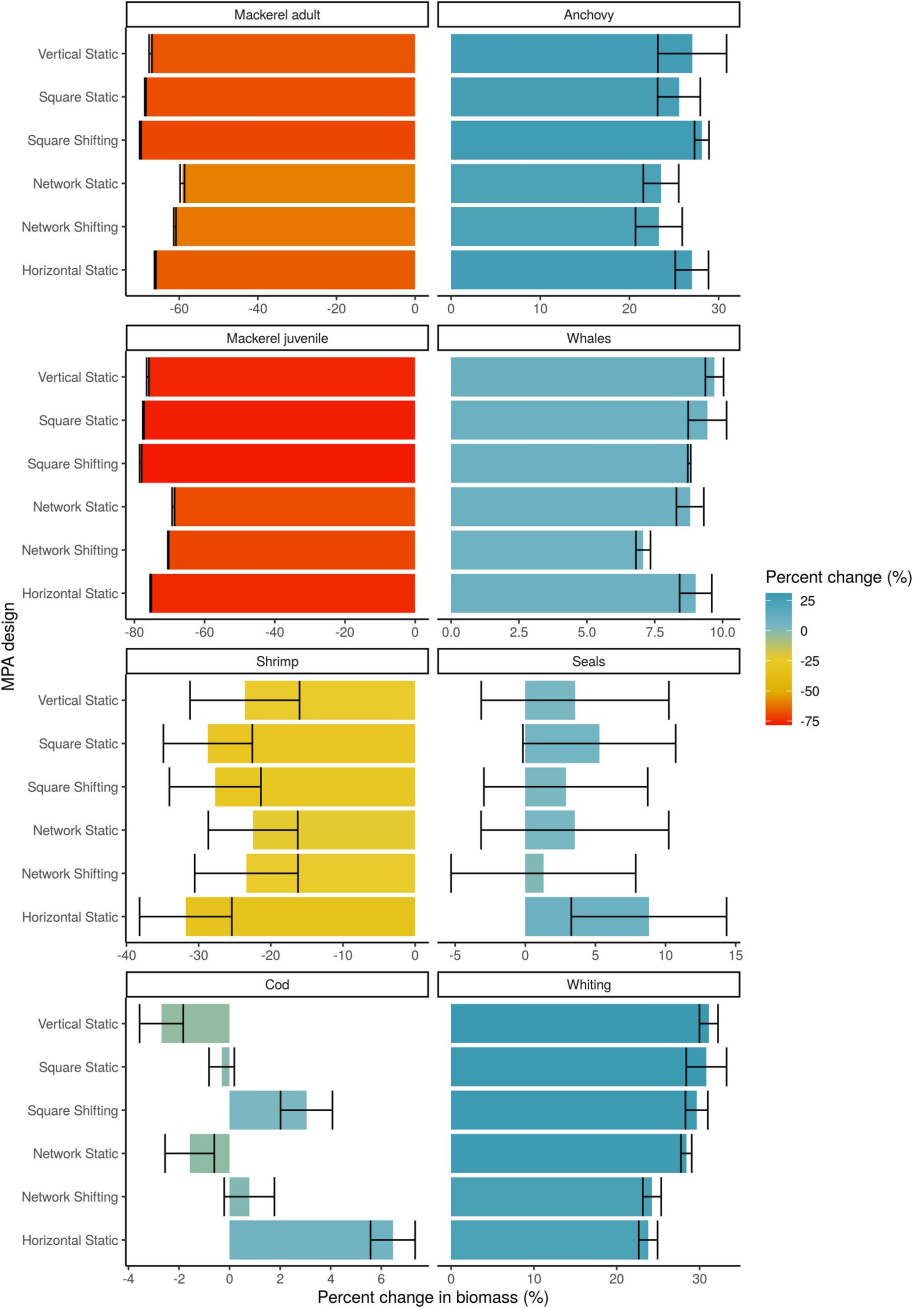
Percent change in biomass of select functional groups compared to no MPA scenario at the end of the 21^st^ century. Error bars represent 95% confidence intervals of the mean percent change.

There are winners and losers for different species and fisheries under the combination of MPA and climate change effects (S8–S24 Figs). The spillover effect of increased catches adjacent to MPAs is observable for those species that thrive within the MPA depending on the MPA design (e.g., for whiting under all MPA designs, S17 Fig). This effect of increasing biomass within the MPA is mediated by the predator-prey interactions that may be affected by whether some species are primarily protected from fisheries. For example, shrimp are negatively affected within the MPAs due to the protection of their predator, whiting. Anchovy and whiting are the two groups that benefit the most compared to the no MPA scenario under all MPA scenarios (Fig 5) with average increases in biomass of 26% and 28%, respectively. The non-network scenarios (the Static Vertical, Horizontal, and Square and the Dynamic Square scenarios) appear to generate more extreme positive outcomes for anchovy, whiting and whales and larger negative outcomes for mackerel (juvenile and adult) and shrimp. This strong negative effect on mackerel is consistent across MPA scenarios (average decline of -65% for adult mackerel).

### Fishery results

**Fig 6:**
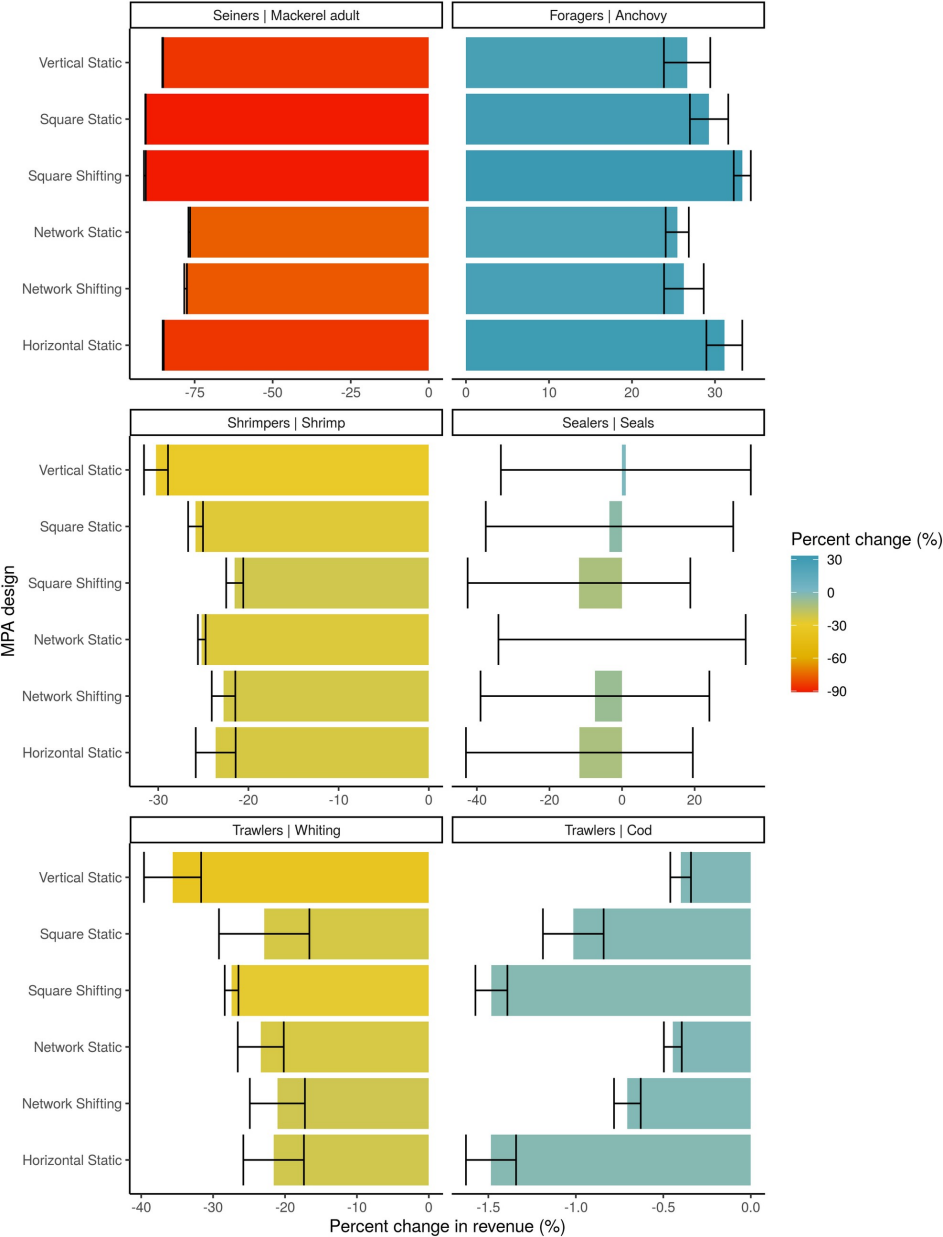
Percent change in revenue of each fishing fleet compared to no MPA scenario at the end of the 21^st^ century. Error bars represent 95% confidence intervals of the mean percent change.

Our results suggest that catches were higher under all MPA scenarios and fisheries revenues were lower under all MPAs compared to the no MPA scenario. This is because catches of high-value species are lower (e.g., shrimp), while low-value species are higher (e.g., anchovy and whiting) (Fig 6). The increase in biomass is followed by an increase in catches with the same fishing effort, thus leading to higher catch per unit effort of these fleets (S8–S24 Figs). Thus, the ‘foragers’ fleet for anchovy and the trawlers for whiting see their catches increase by between 15% and 30% depending on the scenario. In contrast, the mackerel fleet experiences much lower catches, not only because of the area closed to fishing, but also due to the lowered mackerel populations. These changes in the catch profiles under MPA scenarios lead to an aggregate loss in fisheries revenues, even though there are higher total catches. Only the anchovy fleet experiences economic benefits under all the MPA scenarios (Fig 6). The remaining fleets experience almost no change from the no MPA scenario (trawlers for cod) or declines in revenue (all other fleets).

MPAs lead to an aggregate loss in revenue under climate change when compared to a no MPA scenario, which is due to a reduction in fishing area. However, some designs perform better than others (Fig 2). By the end of the 21^st^ century under 4°C warming, the Square Shifting design, followed by the Network Shifting and the Horizontal Static MPA (which are comparable to each other) perform the best in terms of revenue and experience the smallest loss relative to the no MPA scenario. These three designs perform significantly better than the alternatives (Fig 2), but not for all fishing fleets. For example, the Square Shifting MPA has the best result in terms of anchovy fleets (+33%) but the worst result in terms of mackerel fleets (-91%; Fig 6).

## Discussion

Our findings show an increase in biomass in some MPA scenarios (notably Vertical Static, Network Static and Square Shifting), suggesting that, even under climate change, MPAs could provide benefits regionally. However, our model shows that there is no silver bullet design for maintaining ecological functions and the fisheries that depend on them under climate change. Various MPA designs performed relatively similarly to each other in terms of aggregate catch and revenue in comparison to the no MPA scenario, suggesting that MPAs could be beneficial regardless of the design. In terms of fisheries revenue, our MPA designs did not outperform the no MPA scenario under climate change. This finding reinforces the current understanding that trade-offs between species protection and fisheries are often needed [36, 37]. However, our model does demonstrate higher catches on the edges of MPAs that allow fishers to capitalize on the spillover effects of MPAs.

The Vertical and Square Shifting MPA scenarios may mitigate the negative impact of climate change on biomass by protecting species as they migrate to cooler waters because the Vertical MPA covers a larger temperature gradient than the other scenarios, and the Square Shifting MPA moves towards a colder temperature gradient. This may partially address current concerns expressed by [19] of species thermal thresholds being exceeded in current tropical MPAs. However, this will not be true in all cases, where species migration to cooler waters is not poleward [38]. The Network Static MPA increased aggregate biomass, yet it negatively impacted some species more than other MPA designs due to fisheries being able to exploit the spillover effect. In line with this finding, the Network MPAs were the best performing designs for fishery revenues over time.

The potential benefits of dynamic MPAs are likely complicated by the ecological reality of predator-prey interactions and shifting fishing effort. This reinforces earlier findings that the potential benefits of MPAs vary by species [2]. While MPAs are often introduced to protect the marine ecosystem, they appear, in this analysis, to modify a previously fished ecosystem which can exacerbate predator-prey interactions to the point of increasing some populations and dramatically reducing others. An example of this in our model can be seen with mackerel, where even though they were protected from fishing within the MPA, mackerel were still driven to very low biomass levels due to an increase in their predators (whales). Therefore, the idea of MPAs restoring ecosystems to a previous state is complicated by ecological reality. Consequently, MPA design necessitates economic and ecological trade-offs that modify the ecosystem functioning and thus translates into effects on the fishers and others that rely upon these ecosystems [39]. While dynamic MPAs may have ecological benefits [40, 41], they may be difficult in practice near coastal areas where a wide-variety of human activities occur and many people rely on the coastal environment for their livelihoods [42]. This source of conflict is reduced in the High Seas where dynamic MPAs could benefit many threatened species [20].

The results of our study confirm the importance of analyzing predator-prey interactions, and the need for managers to consider these interactions before implementing an MPA. Ecosystem-based management can be particularly helpful instead of single-species based management as the former accounts for species interactions [43, 44]. Our model shows that the implementation of an MPA can shift the balance of an ecosystem in favor of some functional groups over others. To apply this finding, it is important to understand that the establishment of an MPA disrupts a current ecosystem that includes fishing pressure and that the species’ populations will be modified by these spatial restrictions of fishing pressure. These dynamics can be examined by modeling how food webs will respond if heavily fished species or an apex predator is released from fishing pressure [45]. Thus, it is important to design MPAs with specific goals in mind, whether it is the protection of a single species, certain species, or overall protection of the ecosystem.

### Limitations

Our work uses a simple spatial ecosystem model with a sea surface temperature change as a climate change driver. The model allows us to explore trade-offs between social-ecological outcomes resulting from MPA design. This research is not an empirical analysis, thus the results should be interpreted as demonstrative of potential principles and outcomes rather than a strictly literal interpretation. The model as it has been implemented is not subject to other challenges of fisheries and spatial management including non-compliance with no-take areas, especially for dynamically managed areas. The simplified model we adapted does not control for habitat preferences of different functional groups outside temperature. This was done intentionally to isolate the effect of the MPA design from whether the MPA design overlapped with a species habitat. In addition, the temperature effects are driven by changes in sea surface temperature which are more relevant for some functional groups (e.g., anchovy and mackerel) than others (e.g., cod and whiting).

Our models produce spillover effects and the presence of higher catches under all MPA scenarios indicates that spillover compensates for the loss in catch in areas closed to fishing inside MPA boundaries. This finding demonstrates that our parameterized model predicts spillover catches and benefits to fisheries based on the dispersal values used and tested in our sensitivity analysis (see S1 File). The spillover in our model is demonstrated to be non-trivial based on dispersal values used, and these results are robust to alternative spillover values (S5 Fig). However, the strength of the spillover effect is likely to vary by species [2], and this is dependent on the species within the MPA and surrounding region.

Our modeling can help inform the potential benefits and costs of different MPA designs. However, the findings may not be accurate for all ecosystems. Therefore, it is important to consider the specifics of the ecosystem during the MPA design process and to potentially apply this type of modeling framework where possible. This reinforces our understanding of the value of information during MPA design [46].

## Conclusion

Our results suggest that there is no one optimal solution in the face of climate change, but different MPA designs could potentially bring about regional benefits in terms of biomass and catch. In this study, dynamic single MPAs outperformed dynamic or static networks of MPAs. In addition, some species ranges can be maintained over an extended area through MPAs that extend over an environmental gradient. As our study shows, MPA managers must anticipate the effects of released fishing pressure and species interactions on the goals of their MPA. In the face of a changing climate, research that models the potential trade-offs of MPAs for sustainable fisheries management remains relevant on local and global scales.

## Supporting information

S1 Table Basic input parameters for Anchovy Bay Ecopath model [28]. (DOCX)

S2 Table Diet matrix for Anchovy Bay Ecopath model [28]. (DOCX)

S3 Table Ex-vessel prices used for this study. Obtained from [28].
(DOCX)

S4 Table Literature review of price elasticities corresponding to functional groups in this study. Obtained from Asche et al. 2005. Dependent Variable: p:price; q: quantity.
(DOCX)

S5 Table Dispersal values used for Ecospace parameterization. (DOCX)

S6 Table Aggregate results at the end of the century (2090–2099) for all MPA and climate scenarios. Standard deviations included in brackets. All units are in t/km^2^.
(DOCX)

S7 Table Linear model results using aggregate biomass data at the end of the century (2090–2099) for all MPA and 4° warming. Units for ‘Estimate’ are in t/km^2^.
(DOCX)

S8 Table Linear model results using aggregate catch data at the end of the century (2090–2099) for all MPA and 4° warming. Units for ‘Estimate’ are in t/km^2^.
(DOCX)

S9 Table Linear model results using aggregate revenue data at the end of the century (2090–2099) for all MPA and 4° warming. Units for ‘Estimate’ are in t/km^2^.
(DOCX)

S1 Fig Temperature gradient applied to our ecosystem. Red dot indicates the fishing port location.
(TIF)

S2 Fig Static MPA designs. (TIF)

S3 Fig Shifting MPA designs with caption indicating year. (TIF)

S4 Fig Biomass by functional group (total for the ecosystem) under alternative spillover values compared to no MPA scenario at the end of the 21st century. (TIF)

S5 Fig Catch and biomass in cells adjacent to MPAs under alternative spillover values at the end of the 21^st^ century. (TIF)

S6 Fig Percent change in catch by fleet and target functional groups compared to no MPA scenario at the end of the 21^st^ century. Error bars represent 95% confidence intervals of the mean percent change.
(TIF)

S7 Fig Percent change in catch per unit effort (CPUE) by region compared to no MPA scenario at the end of the 21st century. (TIF)

S8 Fig Anchovy biomass at the end of the 21^st^ century. (TIF)

S9 Fig Benthos biomass at the end of the 21^st^ century. (TIF)

S10 Fig Cod biomass at the end of the 21^st^ century. (TIF)

S11 Fig Adult mackerel biomass at the end of the 21^st^ century. (TIF)

S12 Fig Juvenile mackerel biomass at the end of the 21^st^ century. (TIF)

S13 Fig Phytoplankton biomass at the end of the 21^st^ century. (TIF)

S14 Fig Seal biomass at the end of the 21^st^ century. (TIF)

S15 Fig Shrimp biomass at the end of the 21^st^ century. (TIF)

S16 Fig Whale biomass at the end of the 21^st^ century. (TIF)

S17 Fig Whiting biomass at the end of the 21^st^ century. (TIF)

S18 Fig Zooplankton biomass at the end of the 21^st^ century. (TIF)

S19 Fig Anchovy catch at the end of the 21^st^ century. (TIF)

S20 Fig Shrimp catch at the end of the 21^st^ century. (TIF)

S21 Fig Adult mackerel catch at the end of the 21^st^ century. (TIF)

S22 Fig Whiting catch at the end of the 21^st^ century. (TIF)

S23 Fig Cod catch at the end of the 21^st^ century. (TIF)

S24 Fig Seal catch at the end of the 21^st^ century. (TIF)

S1 File Supplemental text. (DOCX)
