# Supplementary material for: Shifting seas, shifting boundaries: Dynamic marine protected area designs for a changing climate
Source: PLoS One. 2020 Nov 10;15(11):e0241771. doi: 10.1371/journal.pone.0241771 (PMC7654810; doi:10.1371/journal.pone.0241771)
Supplement: S1 Table — (DOCX) [file pone.0241771.s001.docx]

*S1 Table. Basic input parameters for Anchovy Bay Ecopath model (Christensen, 2018).*

| Group name | Hab area (proportion) | Biomass in habitat area (t/km2) | Total mortality (/year) | Production / biomass (/year) | Consumption / biomass (/year) | Ecotrophic Efficiency | Other mortality | Production / consumption | Unassim. consumption | Detritus import (t/km2/year) |
| --- | --- | --- | --- | --- | --- | --- | --- | --- | --- | --- |
| Whales | 1 | 0.0800000 |  | 0.05000 | 9.000000 |  |  |  | 0.2 | 0 |
| Seals | 1 | 0.0609000 |  | 0.15390 | 15.000000 |  |  |  | 0.2 | 0 |
| Cod | 1 | 3.0000000 |  | 0.31000 | 2.580000 |  |  |  | 0.2 | 0 |
| Whiting | 1 | 1.8000000 |  | 0.58100 | 3.100000 |  |  |  | 0.2 | 0 |
| Mackerel juv | 1 | 0.0001567 | 4.0000000 |  | 57.563370 |  |  |  | 0.2 | 0 |
| Mackerel ad | 1 | 1.2000010 | 0.7233334 |  | 4.399998 |  |  |  | 0.2 | 0 |
| Anchovy | 1 | 7.0000000 |  | 1.14875 | 9.130000 |  |  |  | 0.2 | 0 |
| Shrimp | 1 | 0.8000000 |  | 3.00000 |  |  |  | 0.25 | 0.2 | 0 |
| Benthos | 1 |  |  | 3.00000 |  | 0.6 |  | 0.25 | 0.2 | 0 |
| Zooplankton | 1 | 14.8000000 |  | 35.00000 |  |  |  | 0.25 | 0.2 | 0 |
| Phytoplankton | 1 | 9.0000000 |  | 240.00000 |  |  |  |  | 0.0 | 0 |
| Detritus | 1 | 10.0000000 |  |  |  | 0.0 |  |  | 0.0 | 0 |
