# Supplementary material for: Shifting seas, shifting boundaries: Dynamic marine protected area designs for a changing climate
Source: PLoS One. 2020 Nov 10;15(11):e0241771. doi: 10.1371/journal.pone.0241771 (PMC7654810; doi:10.1371/journal.pone.0241771)
Supplement: S2 Table — (DOCX) [file pone.0241771.s002.docx]

*S2 Table. Diet matrix for Anchovy Bay Ecopath model (Christensen, 2018).*

| Group Number | Prey  predator | 1 | 2 | 3 | 4 | 5 | 6 | 7 | 8 | 9 | 10 |
| --- | --- | --- | --- | --- | --- | --- | --- | --- | --- | --- | --- |
| 1 | Whales | 0.0 | 0.00 | 0.00 | 0.00 | 0 | 0.00 | 0 | 0 | 0.0 | 0.0 |
| 2 | Seals | 0.0 | 0.00 | 0.00 | 0.00 | 0 | 0.00 | 0 | 0 | 0.0 | 0.0 |
| 3 | Cod | 0.1 | 0.04 | 0.00 | 0.05 | 0 | 0.00 | 0 | 0 | 0.0 | 0.0 |
| 4 | Whiting | 0.1 | 0.05 | 0.05 | 0.05 | 0 | 0.00 | 0 | 0 | 0.0 | 0.0 |
| 5 | Mackerel juv | 0.0 | 0.00 | 0.00 | 0.00 | 0 | 0.00 | 0 | 0 | 0.0 | 0.0 |
| 6 | Mackerel ad | 0.2 | 0.00 | 0.00 | 0.00 | 0 | 0.00 | 0 | 0 | 0.0 | 0.0 |
| 7 | Anchovy | 0.5 | 0.00 | 0.10 | 0.45 | 0 | 0.55 | 0 | 0 | 0.0 | 0.0 |
| 8 | Shrimp | 0.0 | 0.01 | 0.10 | 0.10 | 0 | 0.00 | 0 | 0 | 0.0 | 0.0 |
| 9 | Benthos | 0.1 | 0.90 | 0.75 | 0.35 | 0 | 0.00 | 0 | 1 | 0.1 | 0.0 |
| 10 | Zooplankton | 0.0 | 0.00 | 0.00 | 0.00 | 1 | 0.45 | 1 | 0 | 0.1 | 0.0 |
| 11 | Phytoplankton | 0.0 | 0.00 | 0.00 | 0.00 | 0 | 0.00 | 0 | 0 | 0.1 | 0.9 |
| 12 | Detritus | 0.0 | 0.00 | 0.00 | 0.00 | 0 | 0.00 | 0 | 0 | 0.7 | 0.1 |
