# Supplementary material for: Shifting seas, shifting boundaries: Dynamic marine protected area designs for a changing climate
Source: PLoS One. 2020 Nov 10;15(11):e0241771. doi: 10.1371/journal.pone.0241771 (PMC7654810; doi:10.1371/journal.pone.0241771)
Supplement: S3 Table — Obtained from [28]. (DOCX) [file pone.0241771.s003.docx]

*S3 Table. Ex-vessel prices used for this study. Obtained from Christensen, 2018.*

| Group name | Sealers ($ /biomass) | Trawlers ($ /biomass) | Seiners ($ /biomass) | Foragers ($ /biomass) | Shrimpers ($ /biomass) |
| --- | --- | --- | --- | --- | --- |
| Seals | 6 |  |  |  |  |
| Cod |  | 10 |  |  |  |
| Whiting |  | 4 |  |  |  |
| Mackerel adult |  |  | 4 |  |  |
| Anchovy |  |  |  | 3 |  |
| Shrimp |  |  |  |  | 20 |
