# Supplementary material for: Shifting seas, shifting boundaries: Dynamic marine protected area designs for a changing climate
Source: PLoS One. 2020 Nov 10;15(11):e0241771. doi: 10.1371/journal.pone.0241771 (PMC7654810; doi:10.1371/journal.pone.0241771)
Supplement: S4 Table — Obtained from Asche et al. 2005. Dependent Variable: p:price; q: quantity. (DOCX) [file pone.0241771.s004.docx]

S4 Table. Literature review of price elasticities corresponding to functional groups in this study. Obtained from Asche et al. 2005. Dependent Variable: p:price; q: quantity.

| Product | Own-Price elasticity | Study | Type of data | Region | Dependent Variable | Functional Group |
| --- | --- | --- | --- | --- | --- | --- |
| Cod | -3.15 | Bell (1968) | Ex. Vessel | USA | p | Cod |
| Cod | -8.33 | Barten and Bettendorf (1989) | Ex. Vessel | Belgium | p | Cod |
| Cod fillets | -0.46 | Tsoa, Schrank and Roy (1982) | Wholesale? | USA | q | Cod |
| Danish peeled shrimp | -0.67 | Myrland og Vassdal (1998) | Trade | UK | q | Shrimp |
| Danish shell-on shrimp | 0.02 | Myrland og Vassdal (1998) | Trade | UK | q | Shrimp |
| Domestic shrimp | -0.45 | Sun (1995) | Trade/Ex. Vessel | USA | q | Shrimp |
| Farm-raised shrimp | -0.34 | Sun (1995) | Trade/Ex. Vessel | USA | q | Shrimp |
| Fat fish (Pelagic) | -1.60 | Burton (1992) | Retail | UK | q | Mackerel ad |
| Frozen cod | -1.06 | Myrland og Vassdal (1998) | Trade | UK | q | Cod |
| Frozen cod blocks | -3.16 | Mazany, Roy and Schrank (1996) | Trade/Ex. Vessel | Canada/USA | q | Cod |
| Frozen cod fillets | -1.89 | Mazany, Roy and Schrank (1996) | Trade/Ex. Vessel | Canada/USA | q | Cod |
| Frozen fillets of cod | -1.22 | Myrland og Vassdal (1998) | Trade | UK | q | Cod |
| Horse mackerel | -1.28 | Wessells and Wilen (1994) | Retail | Japan | q | Mackerel ad |
| Icelandic peeled shrimp | -1.08 | Myrland og Vassdal (1998) | Trade | UK | q | Shrimp |
| Norwegian peeled shrimp | -1.89 | Myrland og Vassdal (1998) | Trade | UK | q | Shrimp |
| Shrimp, Lobster | -1.37 | Wessells and Wilen (1994) | Retail | Japan | q | Shrimp |
| Thai peeled shrimp | -0.26 | Myrland og Vassdal (1998) | Trade | UK | q | Shrimp |
| Whiting | -17.05 | Bell (1968) | Ex. Vessel | USA | p | Whiting |
| Whiting | -7.69 | Barten and Bettendorf (1989) | Ex. Vessel | Belgium | p | Whiting |
| Wild-caught shrimp | -0.57 | Sun (1995) | Trade/Ex. Vessel | USA | q | Shrimp |
| Fat fish (Pelagic) | -1.60 | Burton (1992) | Retail | UK | q | Anchovy |
