# Supplementary material for: Shifting seas, shifting boundaries: Dynamic marine protected area designs for a changing climate
Source: PLoS One. 2020 Nov 10;15(11):e0241771. doi: 10.1371/journal.pone.0241771 (PMC7654810; doi:10.1371/journal.pone.0241771)
Supplement: S5 Table — (DOCX) [file pone.0241771.s005.docx]

*S5 Table. Dispersal values used for Ecospace parameterization.*

| Functional group | Base dispersal rate (km/year) |
| --- | --- |
| Whales | 300 |
| Seals | 300 |
| Cod | 150 |
| Whiting | 100 |
| Mackerel juv | 10 |
| Mackerel ad | 10 |
| Anchovy | 100 |
| Shrimp | 10 |
| Benthos | 10 |
| Zooplankton | 300 |
| Phytoplankton | 300 |
| Detritus | 5 |
