# Supplementary material for: Shifting seas, shifting boundaries: Dynamic marine protected area designs for a changing climate
Source: PLoS One. 2020 Nov 10;15(11):e0241771. doi: 10.1371/journal.pone.0241771 (PMC7654810; doi:10.1371/journal.pone.0241771)
Supplement: S6 Table — Standard deviations included in brackets. All units are in t/km2. (DOCX) [file pone.0241771.s006.docx]

*S6 Table. Aggregate results at the end of the century (2090-2099) for all MPA and climate scenarios. Standard deviations included in brackets. All units are in t/km^2^.*

| Measure | Horizontal static | Network shifting | Network static | Square shifting | Square static | Vertical static | No MPA |
| --- | --- | --- | --- | --- | --- | --- | --- |
| Biomass | 74.2 (0.17) | 74 (0.22) | 74.3 (0.19) | 74.4 (0.16) | 74.1 (0.22) | 74.4 (0.3) | 74 (0.19) |
| Catch | 4.9 (0.04) | 4.8 (0.04) | 4.8 (0.02) | 5 (0.01) | 4.9 (0.04) | 4.8 (0.05) | 4.4 (0.01) |
