# Supplementary material for: Shifting seas, shifting boundaries: Dynamic marine protected area designs for a changing climate
Source: PLoS One. 2020 Nov 10;15(11):e0241771. doi: 10.1371/journal.pone.0241771 (PMC7654810; doi:10.1371/journal.pone.0241771)
Supplement: S7 Table — Units for ‘Estimate’ are in t/km2. (DOCX) [file pone.0241771.s007.docx]

*S7 Table. Linear model results using aggregate biomass data at the end of the century (2090-2099) for all MPA and 4° warming. Units for ‘Estimate’ are in t/km^2^.*

| **Variable** | **Estimate** | **Std. Error** | **t-statistic** | **p-value** |
| --- | --- | --- | --- | --- |
| Intercept | 74.011 | 0.065 | 1136.035 | 0.000 |
| Horizontal Static | 0.185 | 0.092 | 2.009 | 0.049 |
| Network Shifting | -0.012 | 0.092 | -0.132 | 0.896 |
| Network Static | 0.306 | 0.092 | 3.319 | 0.002 |
| Square Shifting | 0.414 | 0.092 | 4.489 | 0.000 |
| Square Static | 0.033 | 0.092 | 0.354 | 0.725 |
| Vertical Static | 0.326 | 0.092 | 3.535 | 0.001 |
