# Supplementary material for: Shifting seas, shifting boundaries: Dynamic marine protected area designs for a changing climate
Source: PLoS One. 2020 Nov 10;15(11):e0241771. doi: 10.1371/journal.pone.0241771 (PMC7654810; doi:10.1371/journal.pone.0241771)
Supplement: S8 Table — Units for ‘Estimate’ are in t/km2. (DOCX) [file pone.0241771.s008.docx]

S8 Table. Linear model results using aggregate catch data at the end of the century (2090-2099) for all MPA and 4° warming. Units for ‘Estimate’ are in t/km^2^.

| **Variable** | **Estimate** | **Std. Error** | **t-statistic** | **p-value** |
| --- | --- | --- | --- | --- |
| Intercept | 4.365 | 0.011 | 392.644 | 0.000 |
| Horizontal Static | 0.585 | 0.016 | 37.184 | 0.000 |
| Network Shifting | 0.470 | 0.016 | 29.867 | 0.000 |
| Network Static | 0.448 | 0.016 | 28.486 | 0.000 |
| Square Shifting | 0.654 | 0.016 | 41.589 | 0.000 |
| Square Static | 0.516 | 0.016 | 32.842 | 0.000 |
| Vertical Static | 0.461 | 0.016 | 29.337 | 0.000 |
