# Supplementary material for: Shifting seas, shifting boundaries: Dynamic marine protected area designs for a changing climate
Source: PLoS One. 2020 Nov 10;15(11):e0241771. doi: 10.1371/journal.pone.0241771 (PMC7654810; doi:10.1371/journal.pone.0241771)
Supplement: S9 Table — Units for ‘Estimate’ are in t/km2. (DOCX) [file pone.0241771.s009.docx]

*S9 Table. Linear model results using aggregate revenue data at the end of the century (2090-2099) for all MPA and 4° warming. Units for ‘Estimate’ are in t/km^2^.*

| **Variable** | **Estimate** | **Std. Error** | **t-statistic** | **p-value** |
| --- | --- | --- | --- | --- |
| Intercept | 18.105 | 0.023 | 800.041 | 0.000 |
| Horizontal Static | -1.635 | 0.032 | -51.102 | 0.000 |
| Network Shifting | -1.631 | 0.032 | -50.952 | 0.000 |
| Network Static | -1.799 | 0.032 | -56.223 | 0.000 |
| Square Shifting | -1.556 | 0.032 | -48.632 | 0.000 |
| Square Static | -1.908 | 0.032 | -59.616 | 0.000 |
| Vertical Static | -2.288 | 0.032 | -71.481 | 0.000 |
