# Supplementary figures and images for: Shifting seas, shifting boundaries: Dynamic marine protected area designs for a changing climate
Source: PLoS One. 2020 Nov 10;15(11):e0241771. doi: 10.1371/journal.pone.0241771 (PMC7654810; doi:10.1371/journal.pone.0241771)

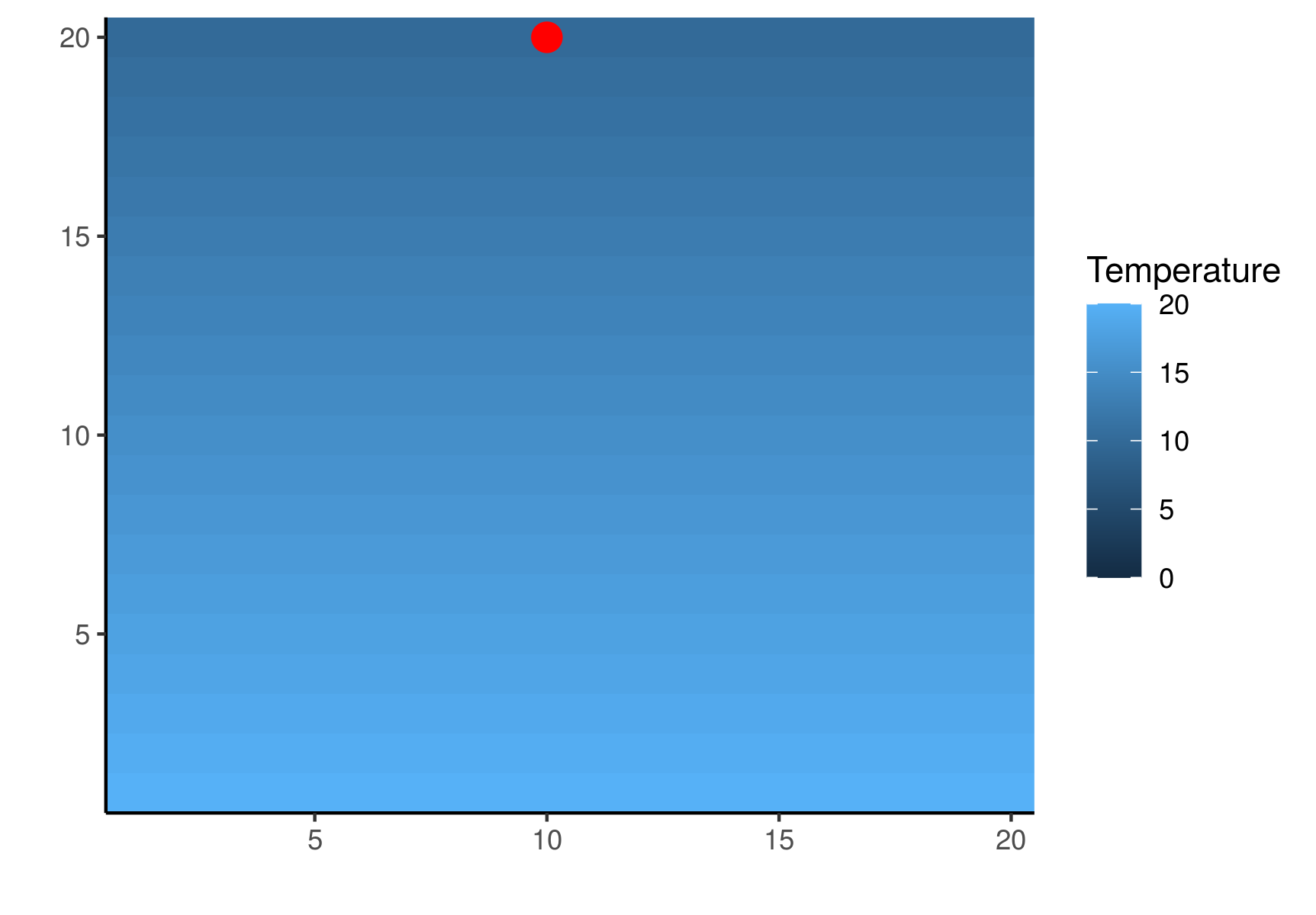

Supplement: S1 Fig — Red dot indicates the fishing port location. (TIF) [file pone.0241771.s010.tif]

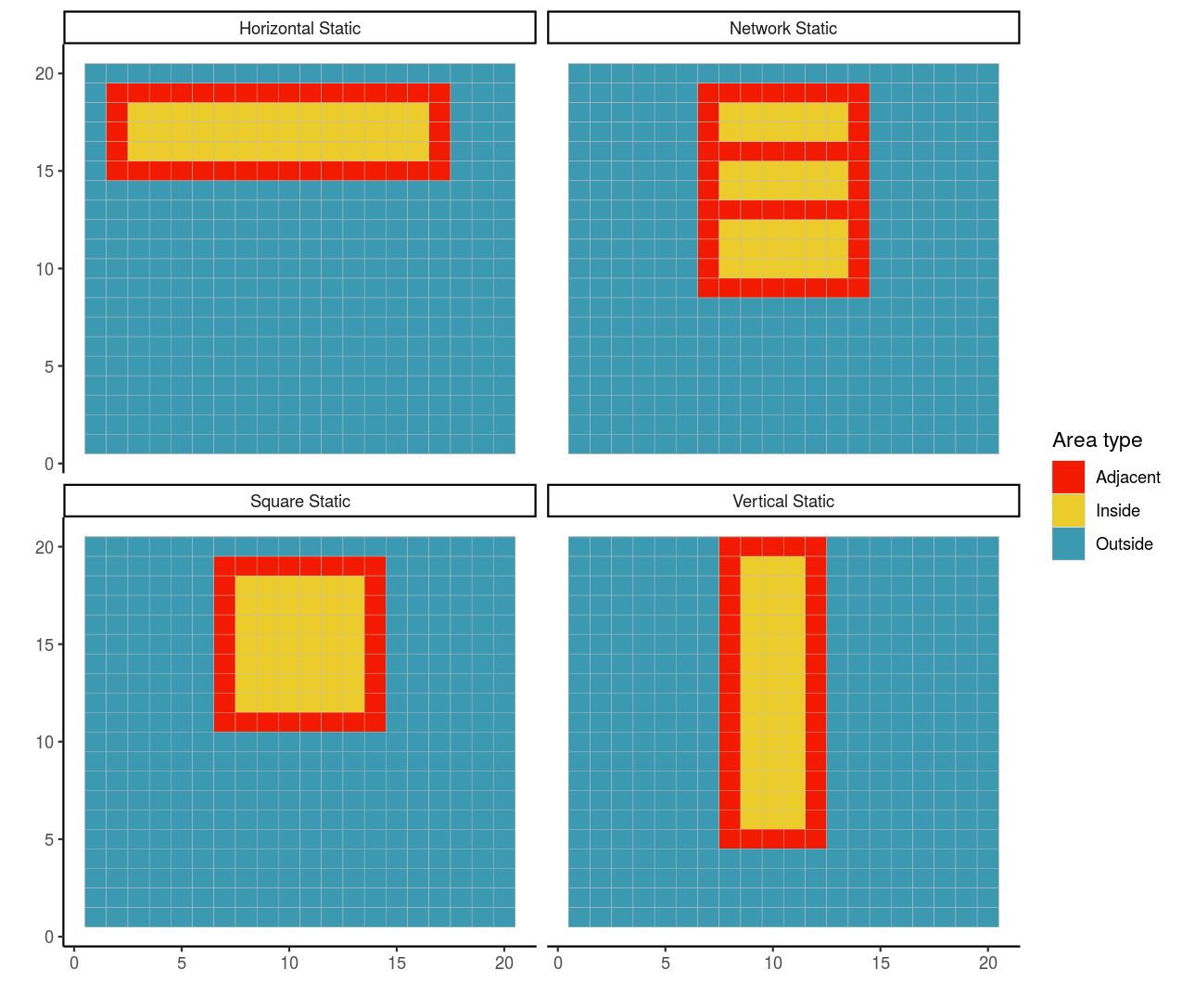

Supplement: S2 Fig — (TIF) [file pone.0241771.s011.tif]

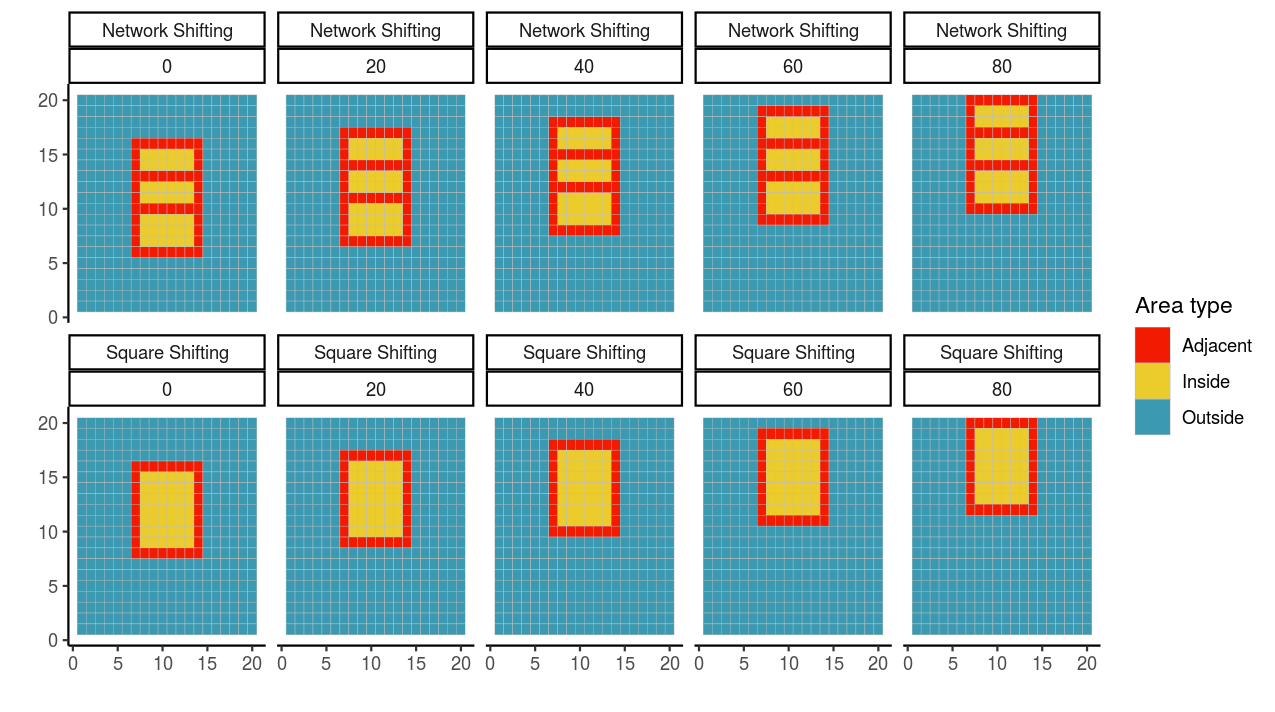

Supplement: S3 Fig — (TIF) [file pone.0241771.s012.tif]

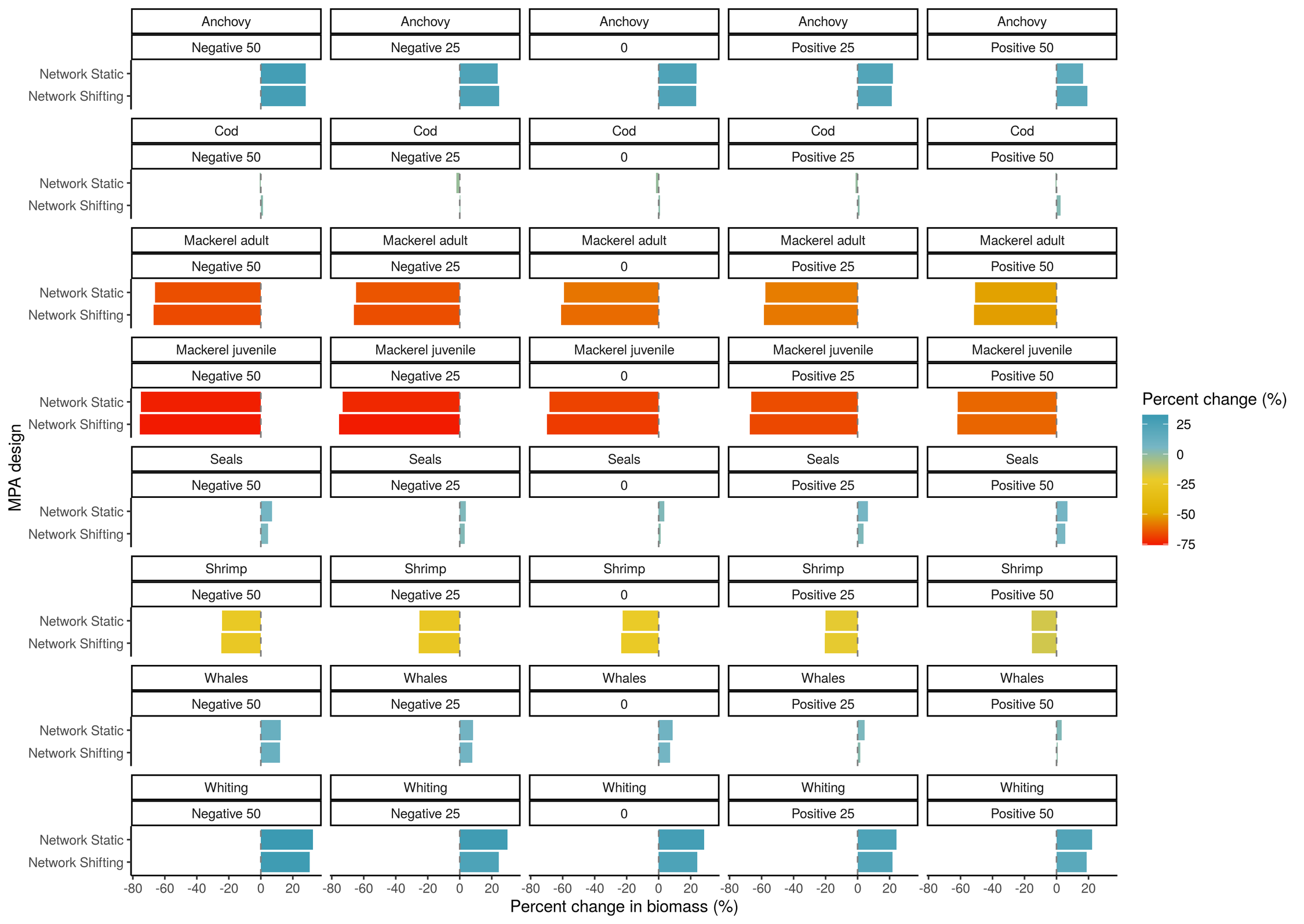

Supplement: S4 Fig — (TIF) [file pone.0241771.s013.tif]

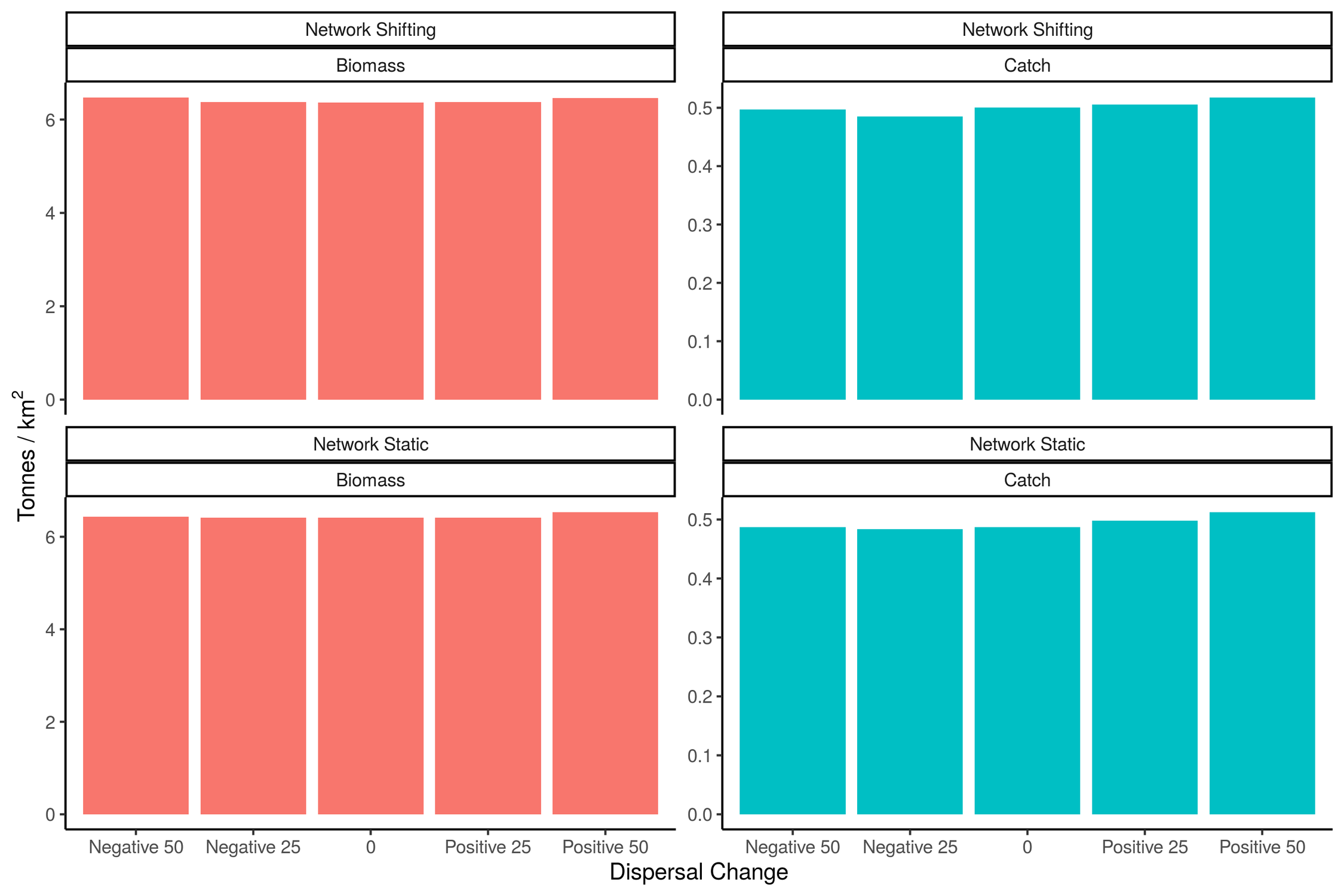

Supplement: S5 Fig — (TIF) [file pone.0241771.s014.tif]

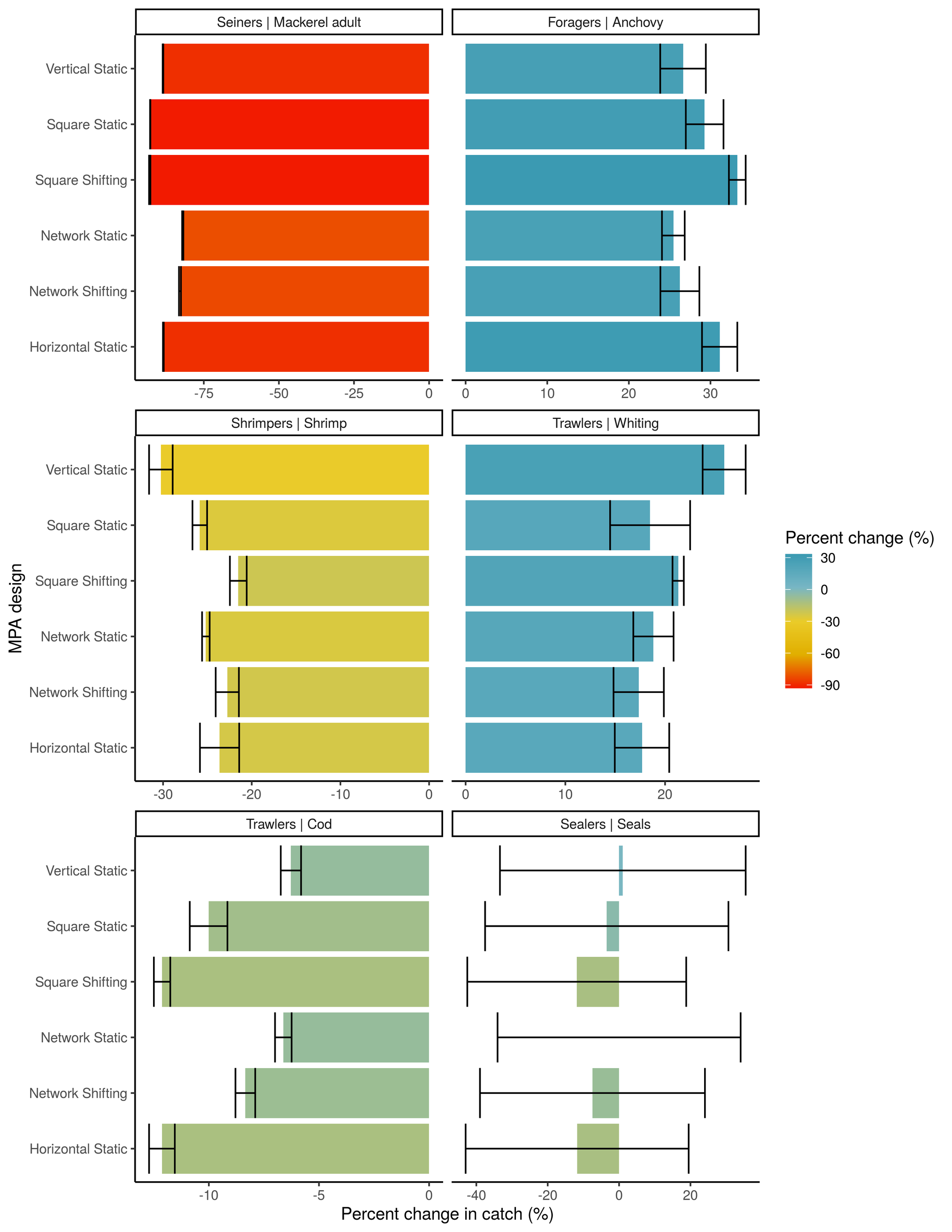

Supplement: S6 Fig — Error bars represent 95% confidence intervals of the mean percent change. (TIF) [file pone.0241771.s015.tif]

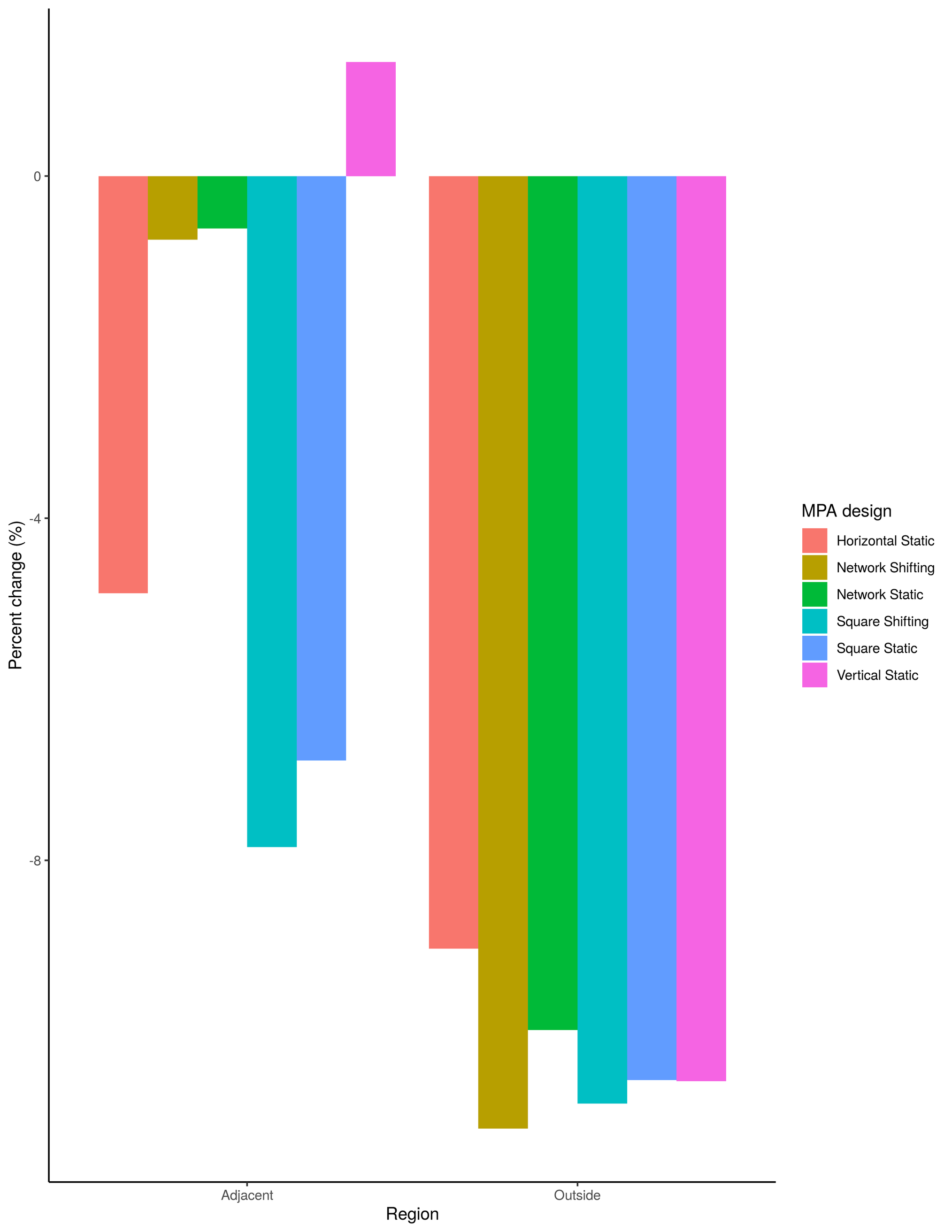

Supplement: S7 Fig — (TIF) [file pone.0241771.s016.tif]

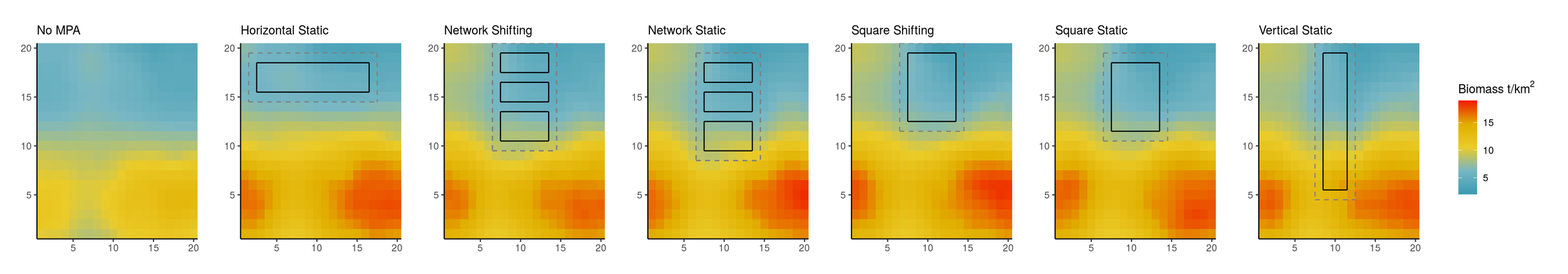

Supplement: S8 Fig — (TIF) [file pone.0241771.s017.tif]

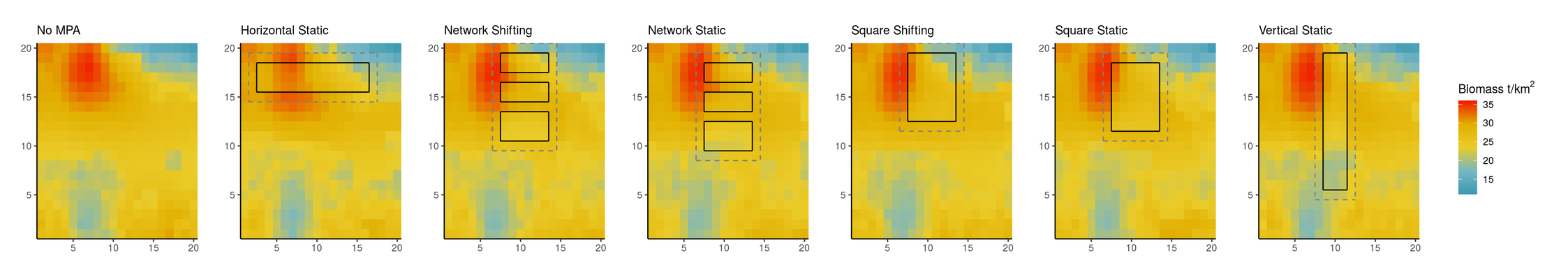

Supplement: S9 Fig — (TIF) [file pone.0241771.s018.tif]

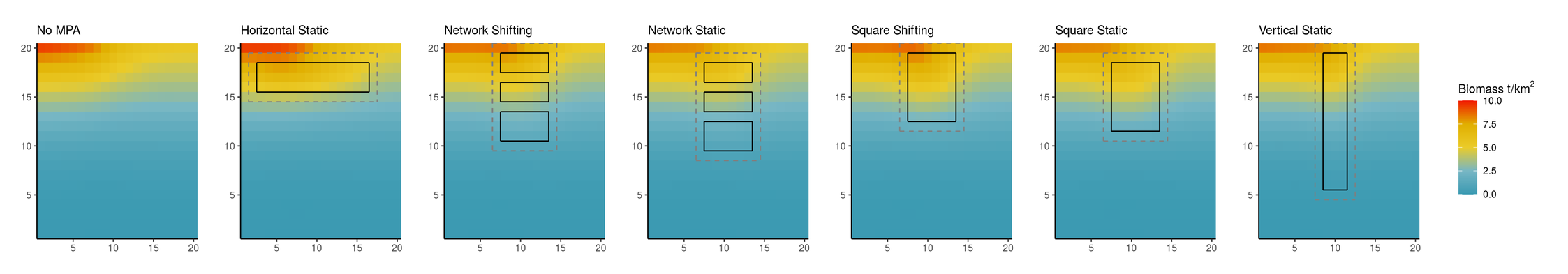

Supplement: S10 Fig — (TIF) [file pone.0241771.s019.tif]

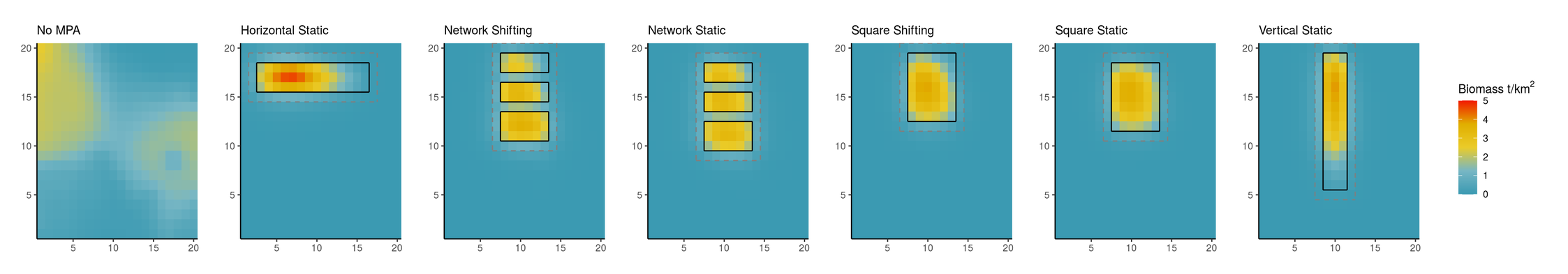

Supplement: S11 Fig — (TIF) [file pone.0241771.s020.tif]

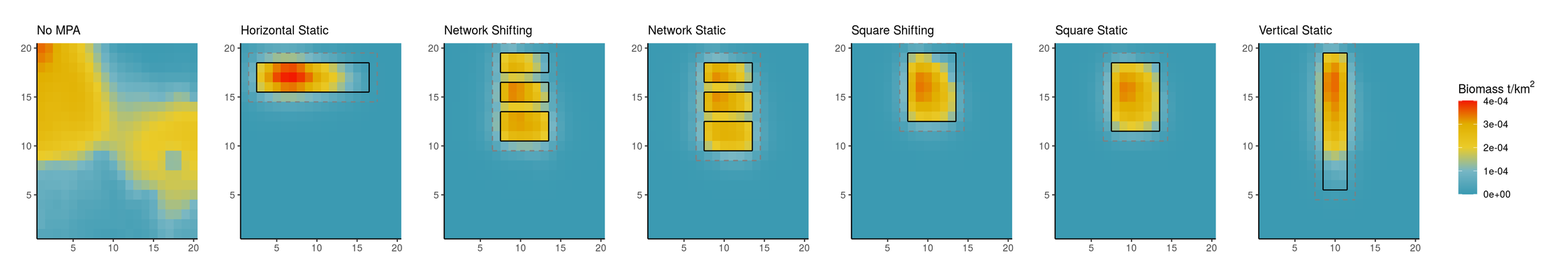

Supplement: S12 Fig — (TIF) [file pone.0241771.s021.tif]

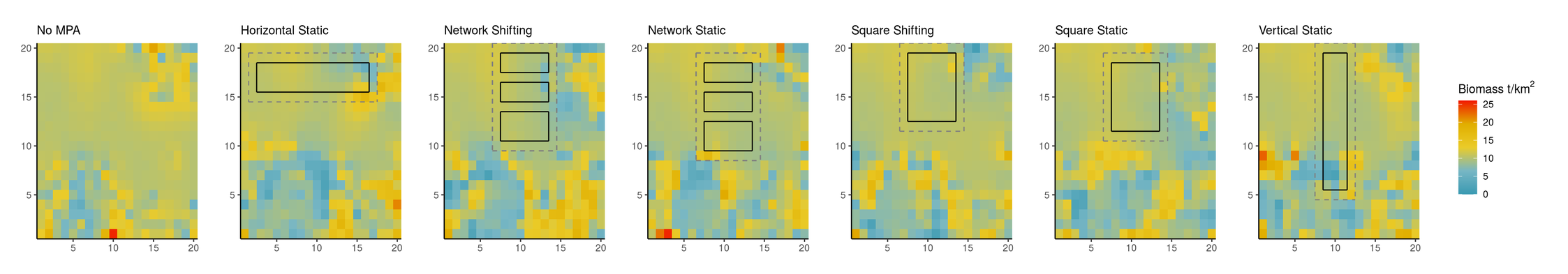

Supplement: S13 Fig — (TIF) [file pone.0241771.s022.tif]

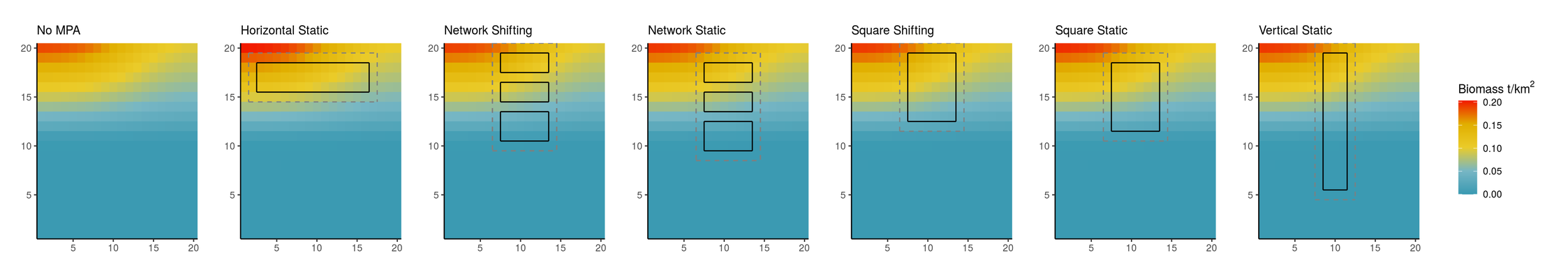

Supplement: S14 Fig — (TIF) [file pone.0241771.s023.tif]

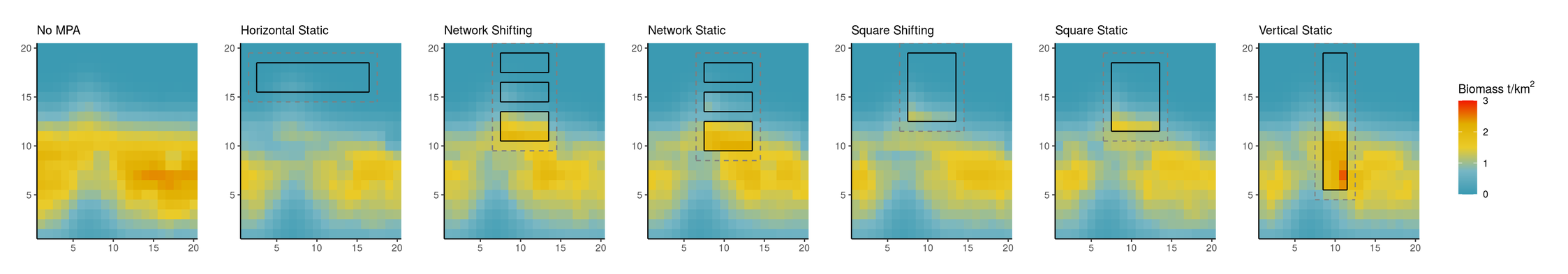

Supplement: S15 Fig — (TIF) [file pone.0241771.s024.tif]

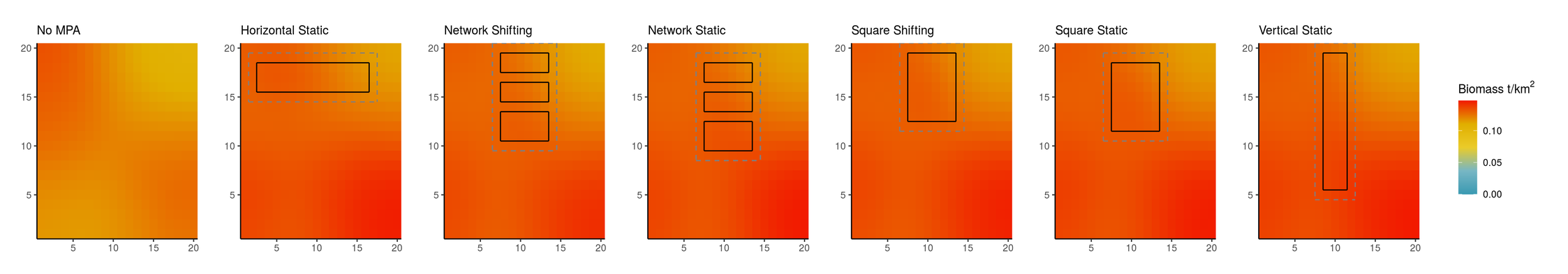

Supplement: S16 Fig — (TIF) [file pone.0241771.s025.tif]

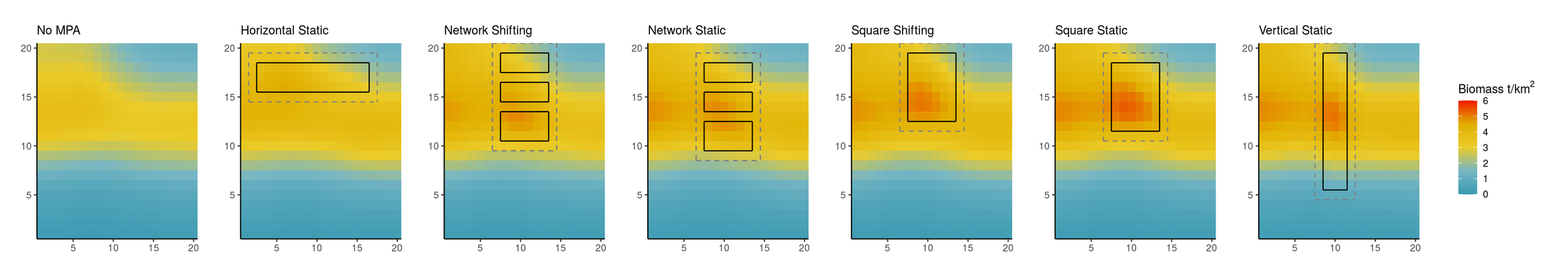

Supplement: S17 Fig — (TIF) [file pone.0241771.s026.tif]

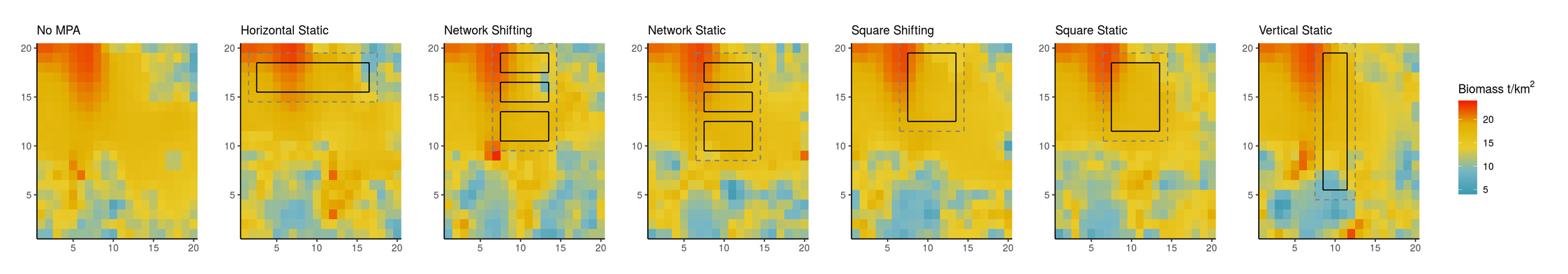

Supplement: S18 Fig — (TIF) [file pone.0241771.s027.tif]

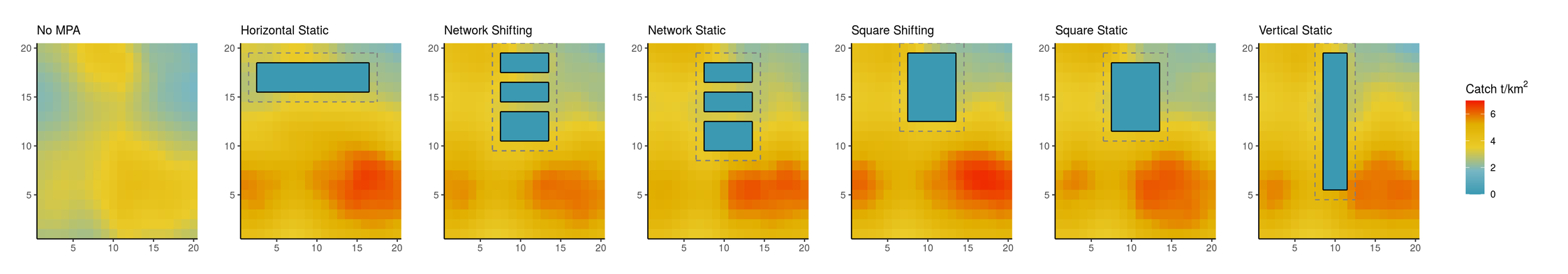

Supplement: S19 Fig — (TIF) [file pone.0241771.s028.tif]

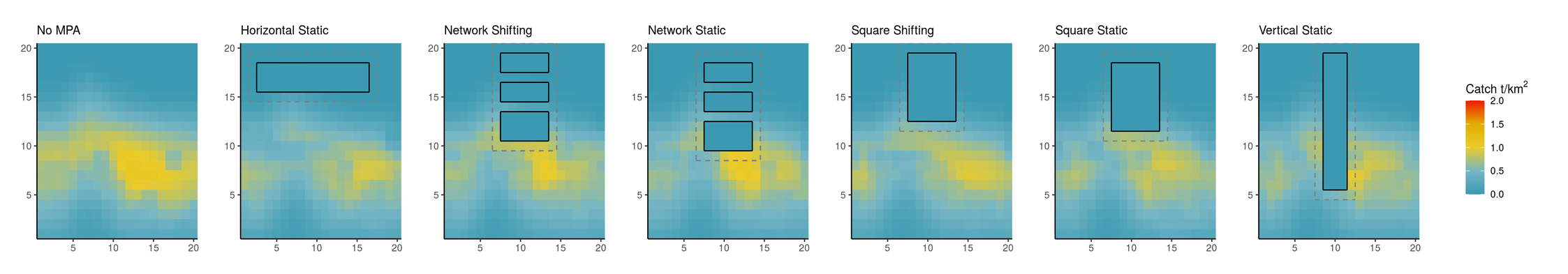

Supplement: S20 Fig — (TIF) [file pone.0241771.s029.tif]

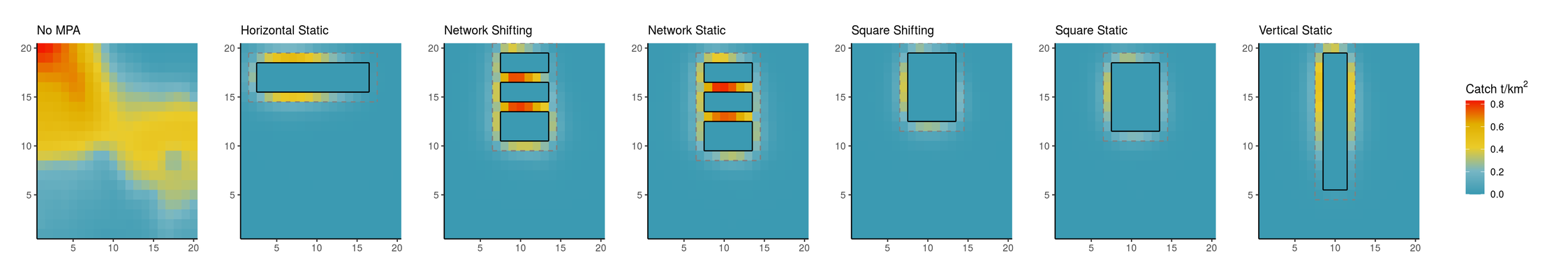

Supplement: S21 Fig — (TIF) [file pone.0241771.s030.tif]

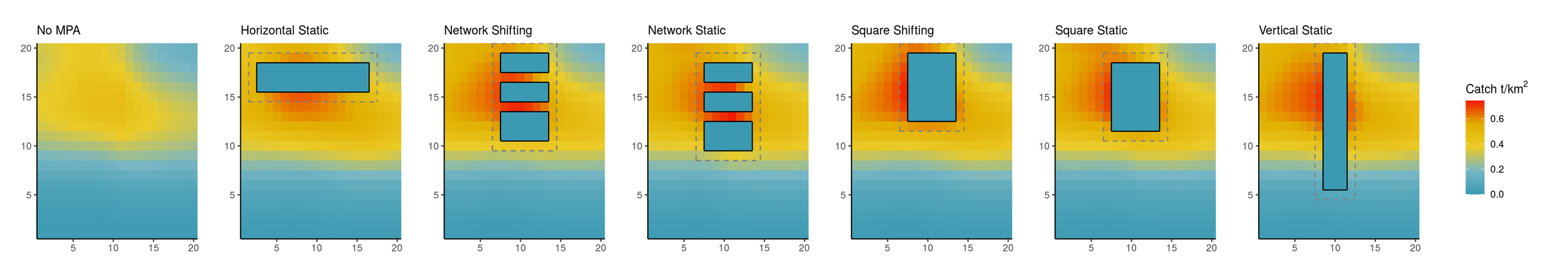

Supplement: S22 Fig — (TIF) [file pone.0241771.s031.tif]

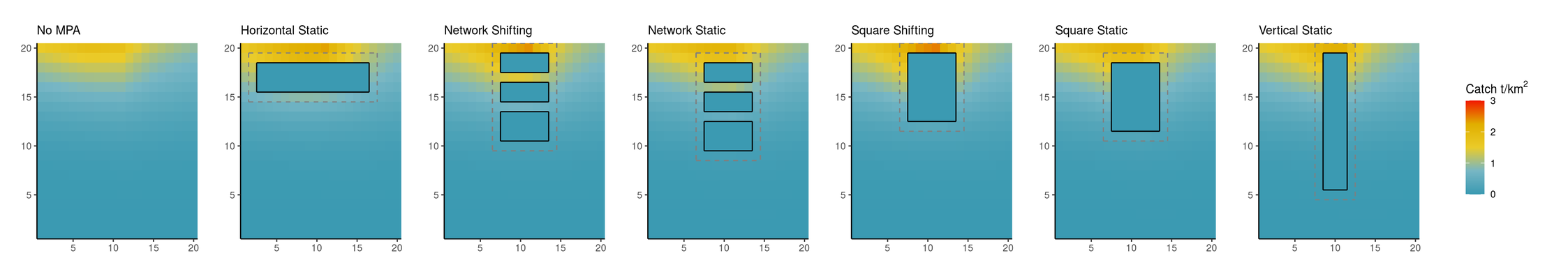

Supplement: S23 Fig — (TIF) [file pone.0241771.s032.tif]

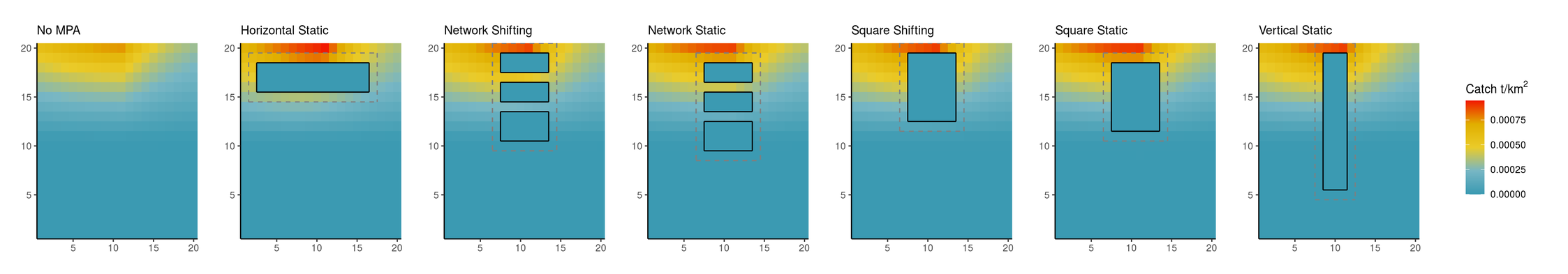

Supplement: S24 Fig — (TIF) [file pone.0241771.s033.tif]
