# Supplementary material for: Shifting seas, shifting boundaries: Dynamic marine protected area designs for a changing climate
Source: PLoS One. 2020 Nov 10;15(11):e0241771. doi: 10.1371/journal.pone.0241771 (PMC7654810; doi:10.1371/journal.pone.0241771)
Supplement: S1 File — (DOCX) [file pone.0241771.s034.docx]

# Supplemental Text

## Supplemental Methods

To ensure the effect we are focusing on is not driven by pre-existing traits related to habitat for foraging, we remove the habitat drivers from functional groups foraging behaviour. The result is that the species are constrained and influenced by environmental (i.e., temperature and primary productivity) drivers rather than limited by habitat type. This included removing the coastal margin of the Ecospace to not limit functional groups from the edge of the area.

An important part of the Ecospace parameterization is the determination of dispersal values. Dispersal determines the average distance a functional group can travel in a year. As it determines how mobile or sessile a functional group is, it is expected to heavily influence the results of marine protected area simulations. The values for similar functional groups were used from (Beattie et al. 2001), and anchovy, benthos, and zooplankton were assumed to have the same values as herring, ’other macrobenthos, and copepods, respectively (Table S5). The dispersal values of all functional groups were modified for the sensitivity analysis.

The fishing fleets had a cost function applied based on distance, and their fishing effort is determined internally through a profit maximizing mechanism. Therefore, they exploit areas of high abundance over areas of low abundance given equal distances. For our specification, we had one port at the Northern end of the habitat halfway through the area (Figure S1).

## Sensitivity analysis

Our sensitivity analysis is carried out by varying the base dispersal rate, which regulates the MPA spillover effect. To estimate the sensitivity to this parameter we varied this value by 25% and 50% in either direction (i.e., lower or higher base dispersal rates). We ran this second sensitivity analysis on three of our MPA designs (no MPAs, network static, and network shifting) under the 4^circ warming scenario.

When we alter the dispersal parameter, we find the results at the functional group level are altered only slightly at the end of the century (Figure S4). In addition, when we look at the area with the most change, we see little variation in catch or biomass levels at the end of the century in cells adjacent to MPAs which theoretically would be most affected by this dispersal parameter (Figure S5).

## Supplemental Results

At the aggregate level, the fishing effort expended in the ecosystem experiences almost no change from the baseline scenario to the MPA scenarios (Figure S7). Effort outside MPA experiences a higher percentage increase than that in adjacent cells, which suggests the density of fish may be higher in adjacent cells, thus lowering effort. However, the effort is spatially redirected based on the cost of fishing (i.e., distance from port) and ‘drawn’ to areas with high biomass. This is apparent when viewing the results of the network MPAs with large edges that can be exploited by fishers. This likely makes these less effective at protecting their biomass, but improves the catch per unit effort in these areas compared to the baseline scenario.

## References

Beattie, Alasdair, Villy Christensen, Ussif Rashid Sumaila, and Daniel Pauly. 2001. “Marine Protected Areas in the North Sea: A Preliminary Bioeconomic Evaluation Using Ecoseed, a New Game Theory Tool for Use with Ecosystem Simulation Ecopath with Ecosim.” University of British Columbia.
